# wpLogicNet: logic gate and structure inference in gene regulatory networks

**DOI:** 10.1093/bioinformatics/btad072

**Published:** 2023-02-15

**Authors:** Seyed Amir Malekpour, Maryam Shahdoust, Rosa Aghdam, Mehdi Sadeghi

**Affiliations:** School of Biological Sciences, Institute for Research in Fundamental Sciences (IPM), Tehran 19395-5746, Iran; School of Biological Sciences, Institute for Research in Fundamental Sciences (IPM), Tehran 19395-5746, Iran; School of Biological Sciences, Institute for Research in Fundamental Sciences (IPM), Tehran 19395-5746, Iran; Wisconsin Institute for Discovery, University of Wisconsin-Madison, Madison, WI 53715, USA; Department of Medical Genetics, National Institute for Genetic Engineering and Biotechnology, Tehran 1497716316, Iran

## Abstract

**Motivation:**

The gene regulatory process resembles a logic system in which a target gene is regulated by a logic gate among its regulators. While various computational techniques are developed for a gene regulatory network (GRN) reconstruction, the study of logical relationships has received little attention. Here, we propose a novel tool called wpLogicNet that simultaneously infers both the directed GRN structures and logic gates among genes or transcription factors (TFs) that regulate their target genes, based on continuous steady-state gene expressions.

**Results:**

wpLogicNet proposes a framework to infer the logic gates among any number of regulators, with a low time-complexity. This distinguishes wpLogicNet from the existing logic-based models that are limited to inferring the gate between two genes or TFs. Our method applies a Bayesian mixture model to estimate the likelihood of the target gene profile and to infer the logic gate *a posteriori*. Furthermore, in structure-aware mode, wpLogicNet reconstructs the logic gates in TF–gene or gene–gene interaction networks with known structures. The predicted logic gates are validated on simulated datasets of TF–gene interaction networks from *Escherichia coli*. For the directed-edge inference, the method is validated on datasets from *E.coli* and DREAM project. The results show that compared to other well-known methods, wpLogicNet is more precise in reconstructing the network and logical relationships among genes.

**Availability and implementation:**

The datasets and R package of wpLogicNet are available in the github repository, https://github.com/CompBioIPM/wpLogicNet.

**Supplementary information:**

Supplementary data are available at *Bioinformatics* online.

## 1 Introduction

Understanding the complex processes of organ development, disease progression and aging relies on the discovery of gene regulation mechanisms. Gene regulatory networks (GRNs) consist of the regulatory interactions between genes and regulators. Since proteins, which play a vital role in biological processes, are encoded by genes, the interpretation of GRNs is crucial in comprehending the biological process (Jiang and Pugh, 2009). Reconstruction of GRNs is a reverse engineering technique that uses gene expression data to discover the underlying network of gene–gene interactions. The emergence of high-throughput technologies, such as microarrays and next-generation sequencing, has made it possible to investigate the expression of thousands of genes simultaneously. The development of computational methods to reconstruct GRNs is regarded as one of the most essential goals in system biology research.

Although several computational methods have been developed for GRN reconstruction, the research on logical connections has been mostly overlooked (Kotiang and Eslami, 2020; Pušnik *et al.*, 2022). Well-known logic-based models, such as Boolean (Alizad-Rahvar and Sadeghi, 2018; Barman and Kwon, 2017; Wang *et al.*, 2015) or multi-state logic (Collombet *et al.*, 2017; Schlatter *et al.*, 2009), use discrete gene expression data to interpret GRN structure, but some genetic information may be lost during the data discretization process. Moreover, regulatory logics applied in binary variables are insufficient to describe dynamic gene expression characteristics (Yan *et al.*, 2017), and Boolean-based models cannot reconstruct all regulatory interactions using only transcriptomic data from single perturbation studies (Alizad-Rahvar and Sadeghi, 2018). In these studies, gene regulation is described by a logic gate among cooperating regulatory genes (RGs) or transcription factors (TFs) as input variables, to regulate the target gene expressions as output from the gate. For instance, Loregic applies Boolean logics to infer logic gates between two TFs, in controlling their targets (Wang *et al.*, 2015). Fuzzy logic models for interpreting networks based on continuous gene expressions have been proposed (Aldridge *et al.*, 2009; Morris *et al.*, 2011) to address the drawbacks of Boolean and multi-state logics. These techniques, however, are constrained in that they require *a priori*-specified structural knowledge.

LogicTRN is a tool to infer the regulatory relationships between two TFs by integrating information from cis-regulatory elements and transcriptional kinetics (Yan *et al.*, 2017). In LogicTRN, to reduce the time-complexity, only pair-wise relationships between TFs are modeled. LogicNet is another logic-based approach that proposed probabilistic continuous (PC) logic, to reconstruct GRNs from continuous gene expression data (Malekpour *et al.*, 2020). It is a tool for the simultaneous inference of directed edges and logic gates among RGs, without requiring prior knowledge. Considering *p* genes in the network and gates with up to *k* regulators, LogicNet reaches a complexity of $O(2^{2^{k}}p^{k})$, for $k\leq \frac{p}{2}$, per target gene. This high level of computational complexity is a challenge for evaluating candidate RG sets, specifically when the target is regulated by several genes (large *k*).

This study aims to address the shortcomings and restrictions of existing logic-based methods, by introducing Weighted Partitioned LogicNet (wpLogicNet) to infer gene–gene interactions and logic gates among a reasonable number of RGs from continuous gene expressions, accurately and quickly. In a GRN with *p* genes, each gene is considered as a target, while the remaining p−1 genes are evaluated as its potential RGs. Each RG can activate or inhibit the target gene (Touré *et al.*, 2021). Thus, for *k* regulators, there are $2^{k}$ distinct (logic) combinations of RGs, indicating the cooperative (AND) relationships among them. These logic combinations can be displayed as distinct partitions in a Venn diagram. wpLogicNet assumes that each target profile observation is generated from one of these logic combinations with a prior probability. It then applies a Bayesian mixture model to estimate the likelihood of the target gene profile and updates prior probabilities using the expectation–maximization (EM) algorithm. The logic gate, as the union of logic combinations, with a significant likelihood is used to predict the regulatory relationships among RGs, in controlling their target. While LogicNet calculates $2^{2^{k}}$ likelihoods per candidate RG set of size *k*, wpLogicNet applies a Bayesian model to infer the logic gate *a posteriori* and requires only one likelihood calculation for this aim, reaching a time-complexity of $O(p^{k})$, per target gene.

Simulated and real datasets are used to quantitatively evaluate the performance of wpLogicNet. Real microarray gene expressions are obtained from the SOS DNA-repair response pathway in *Escherichia coli* (Ronen *et al.*, 2002; Shen-Orr *et al.*, 2002), and simulated data are obtained from the Dialogue for Reverse Engineering Assessments and Methods3 (DREAM3) (Marbach *et al.*, 2010; Prill *et al.*, 2010). The performance of wpLogicNet is benchmarked against Loregic, for the logic gate inference in the structure-aware mode, and five well-known methods GENIE3 (Huynh-Thu *et al.*, 2010), Narromi (Zhang *et al.*, 2013), KBOOST (Iglesias-Martinez *et al.*, 2021), CNMIT (Aghdam *et al.*, 2015) and LogicNet (Malekpour *et al.*, 2020), for learning directed GRN structures. Results on simulation and real datasets show that wpLogicNet outperforms other tools in learning logic gates and directed GRN structures.

## 2 Materials and methods

**Fig. 1.**
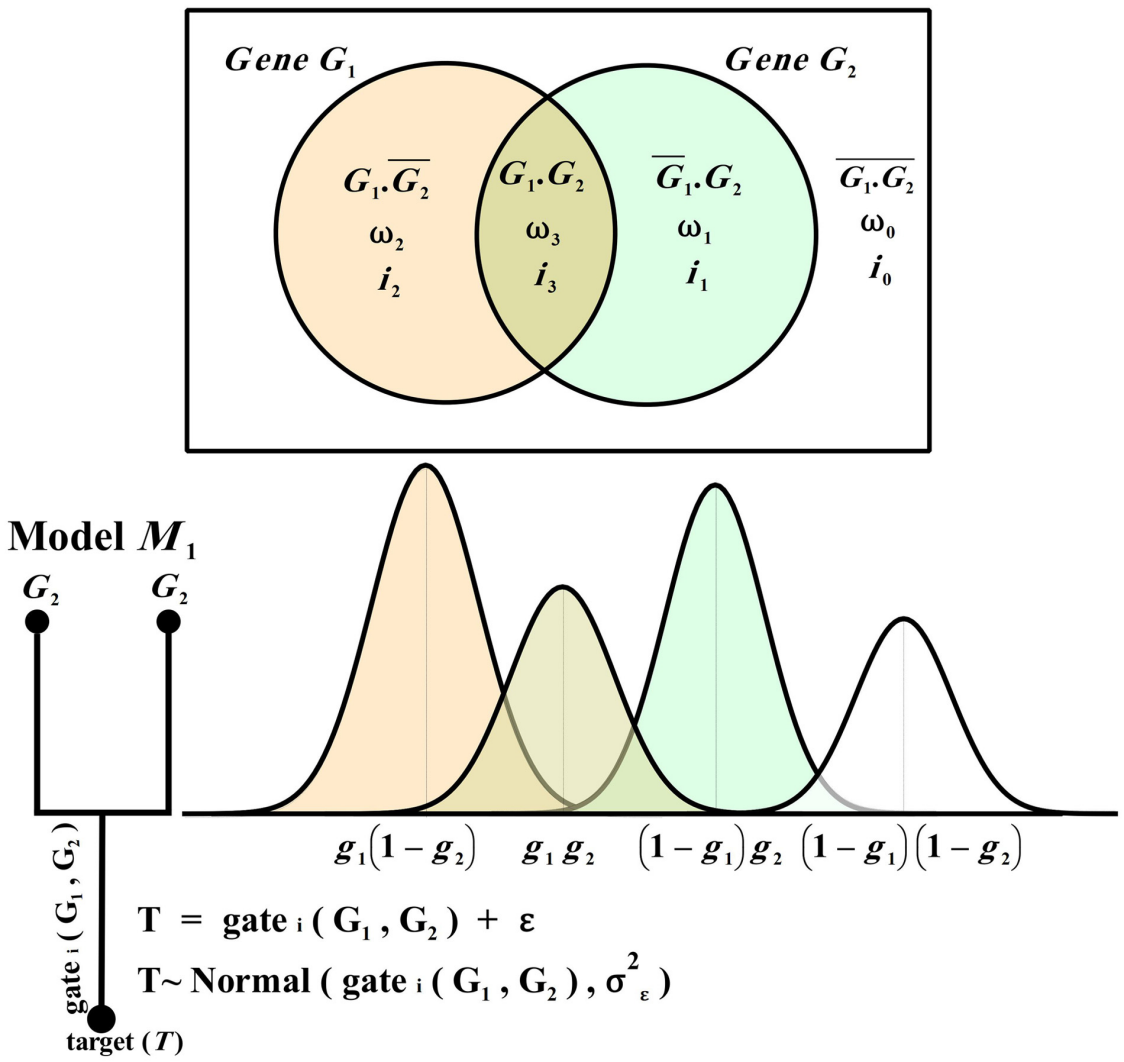
The wpLogicNet’s workflow for two RGs $G_{1}$ and $G_{2}$, *k *=* *2, in controlling their target gene (T), each partition in Venn diagram represents a distinct logic combination, i.e. $G_{1}\cdot G_{2},\;G_{1}\cdot \bar{G_{2}},\;\bar{G_{1}}\cdot G_{2},\;\text{or}\;\bar{G_{1}}\cdot \bar{G_{2}}$, between RGs, with respective outputs, i.e. $g_{1}g_{2},\;g_{1}(1-g_{2}),\;(1-g_{1})g_{2}\;\text{or}\;(1-g_{1})(1-g_{2})$. These outputs define location parameters for Normal densities to approximate the likelihood of generating each sample of *T* under distinct logic combinations, as shown in the lower panel. Indeed, each logic combination contributes a non-binary (location) parameter to the probability of generating a sample of *T* from it, i.e. its activity in the logic gate. Moreover, depending on $g_{1}$ and $g_{2}$ values, locations of densities change in a continuous (0,1) interval and densities may not appear in the same order, as shown above. $\mathrm{gat}e_{i}(G_{1},G_{2})$ defines the logic gate *i* between RGs in controlling *T* and it corresponds to a set of binary variables $i_{v}$, for v=0,1,2,3, representing ON or OFF state of logic combination *v* in generating a sample of *T*. In a gate with multiple ON logic combinations, one is chosen to generate a sample of *T*, i.e. each sample of *T* is normally distributed around the output from the logic combination that generated the sample. The prior probability (weight $\omega_{v}$) of the logic combination *v* in generating a sample, is estimated by the gene expression data in the EM algorithm and is used to infer $i_{v}$’s ON or OFF state *a posteriori*

Given a gene as the target (*T*), wpLogicNet infers its regulators and logic gates, which control or regulate the target gene profiles. Considering $\left\{G_{1},\ldots ,\;\;G_{k}\right\}$ as a candidate set of RGs, each having an activatory or inhibitory effect on *T*, there are $2^{k}$ distinct *logic combinations*, as indicated by different Venn diagram partitions. In Figure 1, for two RGs, $G_{1}$ and $G_{2}$, there are $2^{2}$ distinct logic combinations, $\left\{G_{1}\cdot G_{2},\;G_{1}\cdot \bar{G_{2}},\;\bar{G_{1}}\cdot G_{2},\;\bar{G_{1}}\cdot \bar{G_{2}}\right\}$, each representing a different partition, where ‘·’ stands for AND relationships between genes and $\bar{G_{1}}$ stands for NOT($G_{1}$), representing the inhibitory effect of $G_{1}$ on the target profile. Let $i_{v}$ represents the ON (active) or OFF state of the vth logic combination in regulating target profiles, defined below as:

(1) $$\begin{array}{c} i_{v}=\{\begin{array}{cc} 1 & \mathrm{vth}\;\text{logic combination is ON}\\ 0 & o.w. \end{array} \end{array}.$$

A binary vector $\left[i_{2^{k}-1},\ldots ,i_{1},i_{0}\right]$ represents the ON or OFF state of $2^{k}$ logic combinations. Each vector represents one *logic gate*, as determined by a binary to decimal conversion function $i=\sum_{v=0}^{2^{k}-1}2^{i_{v}}i_{v}$. In Figure 1, with two RGs, i=6 corresponds to the binary vector $\left[i_{3},i_{2},i_{1},i_{0}]=[0,1,1,0\right]$, representing logic gate $\mathrm{XOR}(G_{1},G_{2})=G_{1}\cdot {\bar{G}}_{2}\cup {\bar{G}}_{1}\cdot G_{2}$. To be more precise, each logic gate is constructed by considering the union of active logic combinations.

In wpLogicNet, the normalized gene profile level is in the (0,1) interval, which defines the gene activation probability. See Supplementary Figure S1 for the normalization method. The output generated from a logic combination is calculated with PC logic. As Table 1 shows, the PC logic ${\bar{G}}_{1}$, is expressed with $1-g_{1}$, where the lowercase letter $g_{1}$ denotes the expression level of gene $G_{1}$. Moreover, the output generated from each logic combination (AND relationship among regulators), is regarded as the product of expression levels, e.g. $g_{1}g_{2}$. The 16 possible logic gates between two RGs are summarized in Table 2. The Column 1, *i*, is the logic gate number, and Columns 2–5 show the binary vector $\left[i_{3},i_{2},i_{1},i_{0}\right]$. The logic gate $\mathrm{gat}e_{i}(G1,G2)$ in Column 6 is constructed by considering the union of active logic combinations. The last column shows the possible outputs generated from active logic combinations, in the gate.

**Table 1.** PC logics ‘AND’ and ‘NOT’ for two genes

Logic gate	Logic gate output with PC logic
$\mathrm{NOT}\left(G_{1}\right)\equiv \bar{G_{1}}$	$1-g_{1}$
$\mathrm{AND}\left(G_{1},G_{2}\right)\equiv G_{1}\;\cdot \;G_{2}$	$g_{1}\;g_{2}$

**Table 2.** The 16 possible logic gates between $G_{1}$ and $G_{2}$ in regulating target

*i*	$i_{3}$	$i_{2}$	$i_{1}$	$i_{0}$	$\mathrm{gat}e_{i}\left(G_{1},G_{2}\right)$	Abbreviated notation	Output
0	0	0	0	0	0	*T* = 0	0
1	0	0	0	1	$\bar{G_{1}}\cdot \bar{G_{2}}$	NOR	$\left(1-g_{1})(1-g_{2}\right)$
2	0	0	1	0	$\bar{G_{1}}\cdot G_{2}$	$\bar{G_{1}}\cdot G_{2}$	$(1-g_{1})g_{2}$
3	0	0	1	1	$\bar{G_{1}}\cdot G_{2}\cup \bar{G_{1}}\cdot \bar{G_{2}}$	$\bar{G_{1}}$	$(1-g_{1})g_{2}$ or $\left(1-g_{1})(1-g_{2}\right)$
4	0	1	0	0	$G_{1}\cdot \bar{G_{2}}$	$G_{1}\cdot \bar{G_{2}}$	$g_{1}(1-g_{2})$
5	0	1	0	1	$G_{1}\cdot \bar{G_{2}}\cup \bar{G_{1}}\cdot \bar{G_{2}}$	$\bar{G_{2}}$	$g_{1}(1-g_{2})$ or $\left(1-g_{1})(1-g_{2}\right)$
6	0	1	1	0	$G_{1}\cdot \bar{G_{2}}\cup \bar{G_{1}}\cdot G_{2}$	XOR	$g_{1}(1-g_{2})$ or $(1-g_{1})g_{2}$
7	0	1	1	1	$G_{1}\cdot \bar{G_{2}}\cup \bar{G_{1}}\cdot G_{2}\cup \bar{G_{1}}\cdot \bar{G_{2}}$	NAND	$g_{1}(1-g_{2})$ , $(1-g_{1})g_{2}$ or $\left(1-g_{1})(1-g_{2}\right)$
8	1	0	0	0	$G_{1}\cdot G_{2}$	AND	$g_{1}g_{2}$
9	1	0	0	1	$G_{1}\cdot G_{2}\cup \bar{G_{1}}\cdot \bar{G_{2}}$	XNOR	$g_{1}g_{2}$ or $\left(1-g_{1})(1-g_{2}\right)$
10	1	0	1	0	$G_{1}\cdot G_{2}\cup \bar{G_{1}}\cdot G_{2}$	$G_{2}$	$g_{1}g_{2}$ or $(1-g_{1})g_{2}$
11	1	0	1	1	$G_{1}\cdot G_{2}\cup \bar{G_{1}}\cdot G_{2}\cup \bar{G_{1}}\cdot \bar{G_{2}}$	$\bar{G_{1}}\cup G_{2}$	$g_{1}g_{2}$ , $(1-g_{1})g_{2}$ or $\left(1-g_{1})(1-g_{2}\right)$
12	1	1	0	0	$G_{1}\cdot G_{2}\cup G_{1}\cdot \bar{G_{2}}$	$G_{1}$	$g_{1}g_{2}$ or $g_{1}(1-g_{2})$
13	1	1	0	1	$G_{1}\cdot G_{2}\cup G_{1}\cdot \bar{G_{2}}\cup \bar{G_{1}}\cdot \bar{G_{2}}$	$G_{1}\cup \bar{G_{2}}$	$g_{1}g_{2}$ , $g_{1}(1-g_{2})$ or $\left(1-g_{1})(1-g_{2}\right)$
14	1	1	1	0	$G_{1}\cdot G_{2}\cup G_{1}\cdot \bar{G_{2}}\cup \bar{G_{1}}\cdot G_{2}$	OR	$g_{1}g_{2}$ , $g_{1}(1-g_{2})$ or $(1-g_{1})g_{2}$
15	1	1	1	1	$G_{1}\cdot G_{2}\cup G_{1}\cdot \bar{G_{2}}\cup \bar{G_{1}}\cdot G_{2}\cup \bar{G_{1}}\cdot \bar{G_{2}}$	*T* = 1	Output from any logic combination

*Note*: The ∪ and ‘·’ signs stand for the union and intersection of the sets.

Taking $\mathrm{gat}e_{i}(G_{1},\ldots ,G_{k})$ as the ith logic gate among *k* of RGs, each target profile observation is generated from one active logic combination and approximated by:

(2) $$T=\mathrm{gat}e_{i}(G_{1},\ldots ,G_{k})+\varepsilon ,\;\;\;\;i=0,\ldots ,2^{k}-1.$$

For example, in Table 2, in $\mathrm{gat}e_{6}(G_{1},G_{2})$ with two active logic combinations, every target observation is generated from either $\bar{G_{1}}\cdot G_{2}$ or $G_{1}\cdot \bar{G_{2}}$, with $(1-g_{1})g_{2}$ or $g_{1}(1-g_{2})$ outputs, respectively.

In Equation (2), ε is a noise with Normal distribution, $\varepsilon ∼\text{Normal}\left(0,\sigma_{\varepsilon }^{2}\right)$, where $\sigma_{\varepsilon }^{2}$ is a scale parameter representing the error variance, calculated with error propagation rules (Goodman, 1960), resulting in small values.

In wpLogicNet, the likelihood of a target gene (*T*) is evaluated for all possible RG sets, e.g. for a network with *p* genes, while one gene is considered as a target, there are $\sum_{k=1}^{p-1}\left(\begin{array}{c} p-1\\ k \end{array}\right)$ candidate RG sets. To reduce the time-complexity of the algorithm in evaluating such a high number of candidate RG sets, we applied a Bayesian model to infer the logic gates *a posteriori*. Consider $\omega_{v}$ to be the prior probability of observing $t_{s}$—sth observation of the target profile—under vth logic combination. Therefore, the likelihood function L(T) for the observation vector, $T=(t_{1},\ldots ,\;t_{n})$, of the target gene is:

(3) $$\begin{array}{c} L(T)=\prod_{s=1}^{n}p(t_{s})=\prod_{s=1}^{n}\sum_{v=0}^{2^{k}-1}\omega_{v}p(t_{s}|v)\;\;,\;\sum_{v=0}^{2^{k}-1}\omega_{v}=1\;, \end{array}$$

where $p(t_{s}|v)$ is the probability of observing $t_{s}$ given the vth logic combination. As $t_{s}$ and ε are linearly associated, they follow the same probability density. Then, when using a Normal density for the error; $p(t_{s}|v)∼\text{Normal}(\mathrm{output}\;of\;\mathrm{vth}\;\text{logic combination},\sigma_{\varepsilon }^{2})$. For instance in Figure 1, $p(t_{s}|v=3)∼\text{Normal}(g_{1}^{s}g_{2}^{s},\sigma_{\varepsilon }^{2})$, where $g_{i}^{s}$ is the normalized profile level of sth observation from $G_{i}$. The error term and, consequently, the target profile can follow other truncated densities, such as Logistic, Laplace, or Cauchy, with a domain in the (0,1) interval. The weight parameters $\omega_{v}$ are estimated applying the EM algorithm. With $\Theta =(\omega_{0}\;,\;\omega_{1}\;,\;\ldots ,\;\omega_{2^{k}-1})$, the ‘expected value’ of the log-likelihood function, $Q(\Theta ,\Theta^{m})$, is:

(4) $$\begin{array}{c} \begin{array}{cc} Q(\Theta ,\Theta^{m}) & =\sum_{v=0}^{2^{k}-1}\sum_{s=1}^{n}\log(\omega_{v}p(t_{s}|v))\;p(v|t_{s},\Theta^{m}) \end{array}\\ \begin{array}{c} =\sum_{v=0}^{2^{k}-1}\sum_{s=1}^{n}\log(\omega_{v})\;p(v|t_{s},\Theta^{m})\\ +\sum_{v=0}^{2^{k}-1}\sum_{s=1}^{n}\log(p(t_{s}|v))\;p(v|t_{s},\Theta^{m}), \end{array} \end{array}$$

where $\Theta^{m}=(\omega_{0}^{m}\;,\;\omega_{1}^{m}\;,\;\ldots ,\;\omega_{2^{k}-1}^{m})$ is the parameter estimation in the mth iteration of the EM algorithm and $p(v|t_{s},\Theta^{m})$ is the posterior probability of having $t_{s}$ generated from vth logic combination, under $\Theta^{m}$. By introducing the Lagrange multiplier, lambda, the above equation is maximized with respect to $\omega_{v}$.

(5) $$\begin{array}{c} \frac{\partial }{\partial \omega_{v}}\left[\sum_{v=0}^{2^{k}-1}\sum_{s=1}^{n}\log(\omega_{v})p(v|t_{s},\Theta^{m})+\lambda (\sum_{v=0}^{2^{k}-1}\omega_{v}-1)\right]=0 \end{array}.$$

Which gives the following equation to update $\omega_{v}$, see Supplementary Material for details.

(6) $$\begin{array}{c} \omega_{v}^{m+1}=\frac{1}{n}\sum_{s=1}^{n}p(v|t_{s},\Theta^{m})=\frac{1}{n}\sum_{s=1}^{n}\frac{\omega_{v}^{m}p(t_{s}|v)}{\sum_{v^{\prime }=0}^{2^{k}-1}\omega_{v^{\prime }}^{m}p(t_{s}|v^{\prime })}. \end{array}$$

The parameters are updated for a pre-determined number of iterations or until $\Theta^{m}$’s convergence. The estimated parameter is indicated by $\Theta^{r}$. The likelihood for the observation vector of target gene *T*, given $\Theta^{r}$, is $\prod_{s=1}^{n}\sum_{v=0}^{2^{k}-1}\omega_{v}^{r}p(t_{s}|v)$.

**Significant likelihoods:** Significant likelihoods are selected based on the Bayes factor (BF) (Berger and Pericchi, 2015), which is the ratio of likelihood estimated by wpLogicNet (*L*_1_) and the base likelihood (*L*_0_): $BF=\frac{L_{1}}{L_{0}}$. With *k* as the number of RGs, the base likelihood $L_{0}$ assumes equal weights for all $2^{k}\;$ logic combinations, i.e. $\omega_{v}=\frac{1}{2^{k}}$, $v=0,1,\ldots ,\;2^{k}-1$. Moreover, the location parameter of $p(t_{s}|v)$ is fixed at $\frac{1}{2^{k}}$.

**Guideline for parameter setting:** Before running the EM algorithm, the number of its iterations, the fitting density and its scale parameter, and the BF threshold must be specified by the user. As a guideline, these parameters can be learned by examining the BF rates, i.e. optimal parameters reaching the highest *F*-scores are derived from models with the highest BF rates. For a GRN with *p* genes, the BF rate is introduced as follows:

(7) $$\begin{array}{c} \text{BF rate}=\sum_{i=1}^{p}Z_{G_{i}}{\;\text{log}\;}_{10}{\text{BF}}_{G_{i}} \end{array},$$

$$\begin{array}{c} Z_{G_{i}}=\{\begin{array}{ccc} \text{Number of RGs for}\;G_{i} & \text{if} & {\text{log}\;}_{10}{\text{BF}}_{G_{i}}\geq {\;\text{log}\;}_{10}\text{BF threshold}\\ 0 & \text{if} & {\;\text{log}\;}_{10}{\text{BF}}_{G_{i}}<{\;\text{log}\;}_{10}\text{BF threshold} \end{array} \end{array}.$$

According to this definition, the BF rate is defined as the sum of the BF for different genes, weighted by the number of predicted RGs. As the best-fitting models have a higher likelihood under our model (Fig. 1), we expect a higher BF rate for optimal models.

**Logic gate inference:** Logic gates are inferred from RG sets with the most significant likelihood as measured by the BF. After selecting the RG set, the next step is to infer ON or OFF states of logic combinations. For this purpose, the posterior density of $i_{v}$ given observations from *T* is calculated, i.e. the observation $t_{s}$ is assigned to logic combination $v^{*}$ if:

(8) $$\begin{array}{c} p(v^{*}|t_{s},\Theta^{r})>p(v|t_{s},\Theta^{r})\;\;\text{for}\;\text{all}\;\;v\neq v^{*} \end{array},$$

where, $p(v|t_{s},\Theta^{r})=\frac{\omega_{v}^{r}p(t_{s}|v)}{\sum_{v^{\prime }=0}^{2^{k}-1}\omega_{v^{\prime }}^{r}p(t_{s}|v^{\prime })}$. If the number of assigned samples in a logic combination exceeds a pre-determined threshold, it is considered ON. The final logic gate (top-logic) is constructed by considering the union of ON (active) logic combinations.

**Directed edges inference:** wpLogicNet suggests two modes to infer the input edges of a target gene, top-logics and top-edges. In the top-logics mode, directed edges are inferred from the top-ranked logic gate and corresponding RG set, as described before. There are no input edges for a target gene with no significant logic gate. In the top-edges mode, we use all the logic gates and relevant RG sets with a significant likelihood to infer the input edges to the target gene. Here, after ranking all logic gates in terms of BF significance, each RG is scored by the BF value from the most significant logic gate that includes it. Finally, RGs with scores above a specified threshold are considered as an input edge to the target gene.

**Structure-aware logic gate inference:** In another application, wpLogicNet is used to infer logic gates in GRNs with predefined structures, such as directed edges. wpLogicNet considers both the network structure and gene expression profiles to infer the best-fitting logic gates among known RGs for controlling their target.

**Time-complexity of wpLogicNet:** For a network with *p* genes, and considering logic gates with up to *k* regulators, there are $2^{2}\left(\begin{array}{c} p-1\\ 1 \end{array}\right)+2^{2^{2}}\left(\begin{array}{c} p-1\\ 2 \end{array}\right)+\cdots +2^{2^{k}}\left(\begin{array}{c} p-1\\ k \end{array}\right)$ possible logic gates per target gene, when no prior knowledge of network structure is available. In this formula, $\left(\begin{array}{c} p-1\\ k \end{array}\right)$ is the number of candidate RG sets of size *k* for the potential regulation of the target, and $2^{2^{k}}$is the number of possible logic gates among genes in such RG sets. In our previous tool, LogicNet, the target gene’s likelihood is assessed separately for each possible logic gate among genes in a candidate RG set, reaching a time-complexity of $O(2^{2^{k}}p^{k})$ for $k\leq \frac{p}{2}$ per target gene. wpLogicNet, as proposed in this study, applies a Bayesian model for the logic gate inference and calculates only one likelihood per candidate RG set, i.e. logic gates are inferred *a posteriori* with Equation (8). Then, the total number of evaluated likelihoods in wpLogicNet is $\left(\begin{array}{c} p-1\\ 1 \end{array}\right)+\left(\begin{array}{c} p-1\\ 2 \end{array}\right)+\cdots +\left(\begin{array}{c} p-1\\ k \end{array}\right)$, reaching a complexity of $O(p^{k})$ per target gene. Compared to LogicNet, wpLogicNet considerably speeds up the processing time of candidate RG sets, allowing it to evaluate a larger number of candidate RG sets at the same time.

## 3 Results

### 3.1 Simulation study: logic gate reconstruction in TF–gene network

**Fig. 2.**
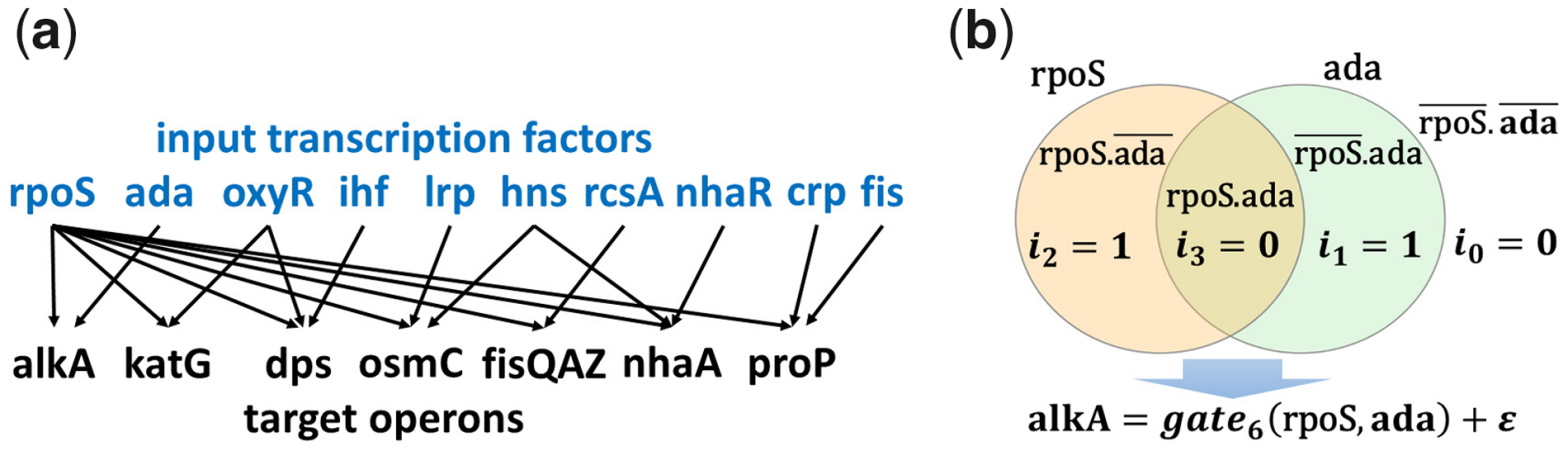
(**a**) The ‘dense overlapping regulons’, i.e. TF–gene network motifs in *E.coli*. (**b**) $\mathrm{gat}e_{6}\left(\mathrm{rpoS},\mathrm{ada}\right)$ representing the output from the logic gate XOR(rpoS, ada) that controls the expression profile of target operon alkA

wpLogicNet’s performance in reconstructing logic gates is evaluated with simulated logic gates from ‘dense overlapping regulons’ (DOR) of *E.coli*. A DOR is defined as a layer of dense overlapping interactions between TFs and their target operons (genes) (Shen-Orr *et al.*, 2002). We use DOR as an example of the TF–gene interaction network to describe wpLogicNet’s application for directed edge and logic gate inference. Figure 2a shows an example of DOR with an input layer of 10 TFs (rpoS, ada, oxyR, ihf, lrp, hns, rcsA, nhaR, crp and fis) that regulate seven operons (alkA, katG, dps, osmC, fisQAZ, nhaA and proP).

To design a specific $\mathrm{gat}e_{i}$, $0\leq i\leq 2^{k}-1$, for each target operon with its specific TFs, $i_{v}$ is generated randomly from 0 (ON state) or 1 (OFF state). Then, 10 TFs are also assigned independently random variables from U(0,1). Given a designed logic gate and TF profiles, we use PC logics (Table 1) to calculate the output generated from, e.g. $\mathrm{gat}e_{i}$.

For each simulated logic gate, continuous gene profile data are generated consistent with Boolean rules. In the $\mathrm{gat}e_{6}(\mathrm{rpoS},\mathrm{ada})$, e.g. that outputs either (1−rpoS)ada or rpoS(1−ada), with low rpoS and high ada expression levels, we choose the logic combination *v *=* *1 to generate (1−rpoS)ada. With high rpoS and low ada, we choose the logic combination *v *=* *2 to generate rpoS(1−ada) from the gate to guarantee a high output in the target approximation. When rpoS and ada are both at low or high levels, generating the output from either logic combinations gives a low expression for target. This process generates data that is consistent with Boolean XOR logics. This simulation process for the alkA operon is illustrated in Figure 2b. The alkA operon is regulated at $\mathrm{gat}e_{i}$ by two TFs (rpoS and ada):

(9) $$\begin{array}{c} \mathrm{alkA}=\mathrm{gat}e_{i}(\mathrm{rpoS},\mathrm{ada})+\varepsilon \end{array}.$$

Given the active logic combinations in the ith gate, possible outputs from $\mathrm{gat}e_{i}$ are rpoSada, rpoS(1-ada), (1-rpoS)ada, or (1-rpoS)(1-ada). For the TF-operon profile simulation, each binary value in $\left[i_{3},i_{2},i_{1},i_{0}\right]$ is sampled randomly from 0 or 1, and rpoS and ada are generated from U(0,1). In Figure 2b, considering $\mathrm{gat}e_{6}$ as simulated gate [0,1,1,0], each sample from alkA could be generated from the logic combination *v *=* *1 or *v *=* *2 with outputs (1-rpoS)ada or rpoS(1-ada), respectively. This value generated from $\mathrm{gat}e_{6}$ will be consistent with Boolean XOR logic, as described above.

The $\varepsilon ∼\text{Normal}(0,\sigma_{\varepsilon }^{2})$ determines the noise generated from Normal density, where $\sigma_{\varepsilon }^{2}$ depends on regulatory TFs and the logic combination generating the observation. For example, if an observation is generated from *v *=* *1, then $\sigma_{\varepsilon }^{2}$ is calculated using Equation (10):

(10) $$\begin{array}{c} \sigma_{\varepsilon }^{2}=\sigma_{\bar{\mathrm{rpoS}}.\mathrm{ada}}^{2}≅ada^{2}\sigma_{\bar{\mathrm{rpoS}}}^{2}+{\left(1-\mathrm{rpoS}\right)}^{2}\sigma_{\mathrm{ada}}^{2} \end{array}.$$

**Fig. 3.**
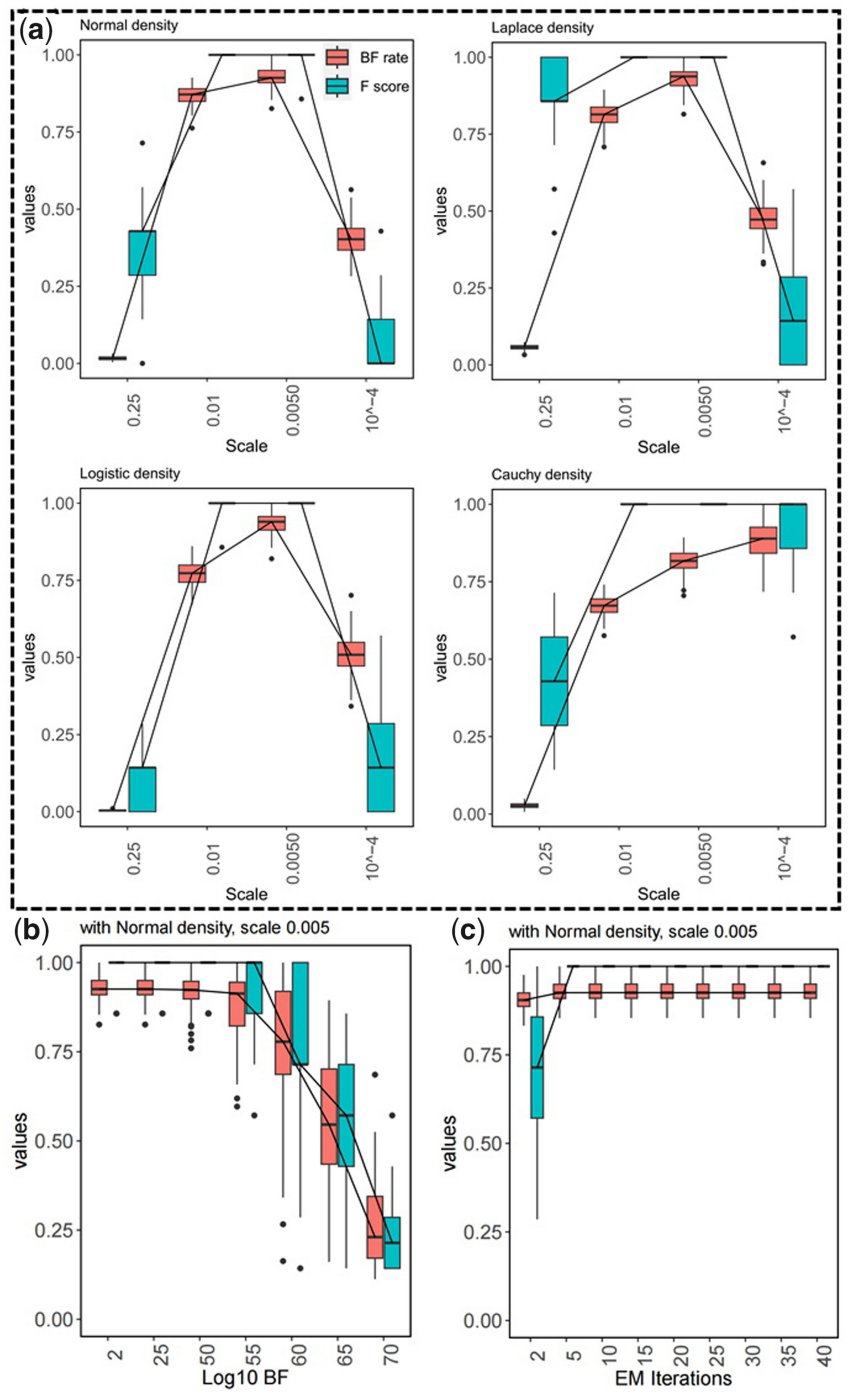
The BF rate (red color) and *F*-scores (blue color) for the logic gate inference in DOR, (**a**) With fitting Normal, Laplace, Logistic and Cauchy densities, over a range of scale parameters $\left\{0.25,0.01,0.005,10^{-4}\right\}$, ${\;\text{log}\;}_{10}\text{BF}$=2 and EM iterations =10. (**b**) With fitting a Normal density, scale =0.005, ${\;\text{log}\;}_{10}\text{BF}$ in the (2,10) interval and EM iterations =10. (**c**) With fitting a Normal density, scale =0.005, ${\;\text{log}\;}_{10}\text{BF}$=2 and EM iterations in the (2,40) interval. With σ=0.01 for all TFs

The variance propagation rule (Goodman, 1960) is utilized to calculate $\sigma_{\bar{\mathrm{rpoS}}.\mathrm{ada}}^{2}$, i.e. the variance of the random variables’ product. Moreover, in our simulation, we consider an identical variance for all TFs, e.g. $\sigma_{\mathrm{ada}}=\sigma_{\bar{\mathrm{rpoS}}}=0.01$. Then, the performance of wpLogicNet in logic gate inference is evaluated on simulated TF-operon profiles. wpLogicNet is implemented for each target operon as follows: (i) we use all 10 TFs as potential regulators for the target operon, (ii) for each candidate regulatory TF set, we evaluate the likelihood of the candidate TF set in approximating the target profiles, and infer the gate *a posteriori* with Equation (8). To reduce the model’s time-complexity, gates with a maximum of four TFs are evaluated. The total number of such gates—per target operon—is $\sum_{i=1}^{k=4}2^{2^{i}}\left(\begin{array}{c} 10\\ i \end{array}\right)=2,303,084.$ For each simulation repeat, only one logic gate is true in controlling the target and all other cases are false. As an example, Figure 2b shows a true positive prediction for $\mathrm{gat}e_{6}(\mathrm{rpoS},\mathrm{ada})$. Predicting any other gate, including those with either a false ON or OFF logic combination state or a false set of predicted regulators, is a false positive. In wpLogicNet, likelihood is calculated based on Normal, Laplace, Logistic and Cauchy densities with a range of scale parameters. (iii) All logic gates (candidate TF sets) are ranked based on likelihood. (iv) The likelihood significance of the logic gate is finally evaluated with BF. Figure 3 shows box-plots for *F*-scores of wpLogicNet in inferring logic gates using 20 samples from TF-operon profiles per simulated gate and 100 repeats of the whole simulation study with σ=0.01 for all TFs. In Figure 3a, wpLogicNet is evaluated with scale parameters $\left\{0.25,0.01,0.005,10^{-4}\right\}$ of fitted densities, ${\;\text{log}\;}_{10}\text{BF}$=2, and EM iterations =10. As shown, the greatest *F*-score, which is almost 1 for the majority of densities is obtained at a scale parameter of 0.005. In Figure 3b, *F*-scores are reported with the scale parameter fixed at 0.005 and ${\;\text{log}\;}_{10}\text{BF}$ in the (2,70) interval and EM iterations =10. It demonstrates that *F*-scores are reduced when ${\;\text{log}\;}_{10}\text{BF}$>55. The sensitivity of the *F*-score to the number of EM iterations is shown in Figure 3c with scale =0.005 and ${\;\text{log}\;}_{10}\text{BF}$=2. Clearly, the algorithm has already converged for iterations >2.

In Supplementary Figure S2, the receiver operating characteristic curve is plotted for logic gate inference in DORs with wpLogicNet. For this purpose, upon ranking the predicted logic gates, more logic gates are included in predictions by gradually reducing the likelihood or BF thresholds. For each threshold, the predicted logic gates are compared with true logics to calculate the false positive rate (FPR) and true positive rate. Using the best-fitting parameters as described above, i.e. Normal density with scale =0.005, ${\;\text{log}\;}_{10}\text{BF}$=2 as a threshold for the significance of predicted logic gate, and EM iterations =10, wpLogicNet reached an area under the curve (AUC) of 0.99 for the logic gate inference. The area under the precision–recall curve (AUPR) is 0.98 as shown in Table 3. In our model, parameters are learned by examining the BF rates, i.e. optimal parameters are derived from models with the highest BF rates. As illustrated in Figure 3a, the optimal scale =0.005 for most densities, also corresponds to the highest BF rate. In Figure 3b, BF rates decrease when ${\;\text{log}\;}_{10}\text{BF}$>55, indicating that ${\;\text{log}\;}_{10}\text{BF}$ should be set to a value <55. In Figure 3c, the optimal value for EM iterations is >2. In Supplementary Table S1, the running time of wpLogicNet is reported considering logic gates with up to *k* candidate TFs. Running times for k=4 (385 candidate TF sets) and k=10 (1023 candidate TF sets) are 14 and 169 s, respectively.

**Table 3.** AUC and AUPR for the logic gate inference in small DORs with 10 TFs and 7 genes, and double sized DORs with 20 TFs and 14 genes, at noise levels σ=0.01, 0.02, 0.03 and sample size =10, 20

		Small DOR		Double sized DOR	
Sample size	σ	AUC	AUPR	AUC	AUPR
10	0.01	0.98	0.91	0.98	0.90
	0.02	0.95	0.82	0.95	0.78
	0.03	0.87	0.45	0.83	0.40
20	0.01	0.99	0.98	0.99	0.95
	0.02	0.99	0.90	0.99	0.89
	0.03	0.96	0.75	0.94	0.74

To measure the sensitivity of the results for each noise level, Table 3 shows the AUC and AUPR of wpLogicNet for other noise levels with σ=0.02,0.03, a sample size =10, 20 in small DORs, and with 10 TFs and 7 genes. See Supplementary Figure S3 for plots to derive the optimal parameters, e.g. scale, for σ=0.02,0.03.wpLogicNet’s performance is also evaluated for double sized TF–gene networks with 20 TFs and 14 genes. We have simulated 100 of these double sized TF–gene networks with distinct edge sets, randomly assigning TFs to target genes for each simulated network. As shown in Table 3, for sample size=20 and σ=0.01, the AUC and AUPR for the double sized DORs are 0.99 and 0.95, respectively.

### 3.2 Structure-aware logic gate inference in the SOS DNA-repair network, with wpLogicNet

**Fig. 4.**
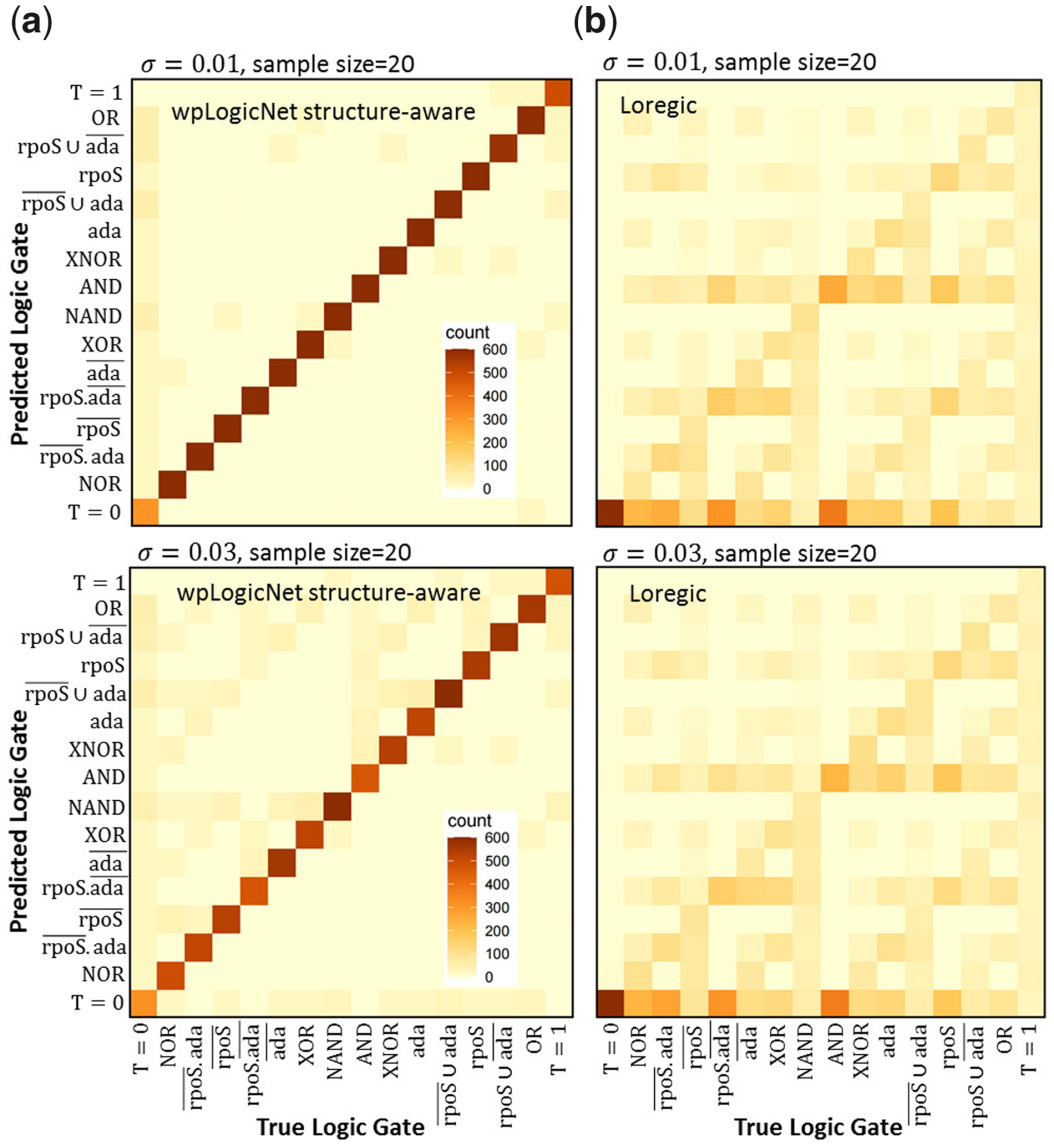
wpLogicNet in structure-aware mode is compared to Loregic. Frequencies (in orange) of the true versus predicted logic gate classes are shown for wpLogicNet (**a**) and Loregic (**b**), for two noise levels σ=0.01, 0.03. For each simulated logic gate, both tools take in the expression profile and directed edges from rpoS and ada –> alkA, to predict the gate. The counts are based on 10 000 simulated logic gates, and 20 samples from TFs-alkA, per gate

wpLogicNet in structure-aware mode infers logic gate by considering the network structure and gene profiles. This applicability of wpLogicNet is compared with Loregic (Wang *et al.*, 2015), which takes in the binarized gene profiles and is restricted to infer 16 logic gates between two TFs or genes, limiting the comparison to *k *=* *2. We applied a similar platform as described in the previous subsection to generate logic gates and data from rpoS, ada and alkA. We have simulated 10 000 logic gates (from 16 possible gates in Table 2), generating 10, 20 samples from TFs-alkA at different noise levels σ=0.01,0.02,0.03 for each logic gate. Then, for logic gate inference, wpLogicNet and Loregic are fed with directed edges from rpoS/ada to alkA and simulated TFs-alkA expression profiles. However, in Loregic, expression profiles are first binarized into 0 and 1 levels. For a sample size =20, and σ=0.01, 0.03, Figure 4 shows the frequencies (in orange) of true versus predicted logic gate classes, for wpLogicNet and Loregic, indicating that there are many more consistencies between predicted and true logic gates in wpLogicNet at both noise levels. However, as Loregic applies the binarized gene profile data, we observed almost no change in its predictions for different noise levels σ=0.01, 0.03, in Figure 4b. In contrast, dependence of the results on the noise levels is more obvious in wpLogicNet. In Figure 4a, for σ=0.03, we observed more inconsistencies between the true and predicted logic gate classes (diagonal counts are in lighter orange) than predictions for σ=0.01. See Supplementary Figure S4 for the results with sample size =10.

In Table 4, AUC and AUPR are calculated for wpLogicNet and Loregic with noise levels σ=0.01, 0.02, 0.03 and sample size =10, 20. This shows that wpLogicNet outperforms Loregic for all noise levels. For example, for σ=0.01 and sample size =20, wpLogicNet with AUC = 0.98 and AUPR = 0.97 is superior to Loregic with AUC = 0.82 and AUPR = 0.60.

**Table 4.** AUC and AUPR for wpLogicNet and Loregic in the logic gate inference, at noise levels σ=0.01, 0.02, 0.03, sample size =10, 20 and 10 000 simulated logic gates

		wpLogicNet, *k* = 2		Loregic	
Sample size	σ	AUC	AUPR	AUC	AUPR
10	0.01	0.94	0.88	0.80	0.44
	0.02	0.94	0.83	0.79	0.43
	0.03	0.93	0.72	0.79	0.42
20	0.01	0.98	0.97	0.82	0.60
	0.02	0.98	0.86	0.82	0.59
	0.03	0.97	0.73	0.81	0.58

wpLogicNet is also applied to infer logic gates given the network structure and the real microarray data of the SOS DNA-repair response pathway in *E.coli* (Bansal *et al.*, 2006; Gardner *et al.*, 2003; Marbach *et al.*, 2010; Prill *et al.*, 2010). The SOS network includes two mediators of the SOS response (lexA and recA), four other RGs (ssb, recF, dinI and umuDC) involved in the SOS response and the three sigma factor genes (rpoD, rpoH and rpoS) whose regulation plays important role in the SOS response. For this SOS network with nine genes, there are two real microarray data (SOS1 and SOS2). SOS1 contains 9 samples (Gardner *et al.*, 2003) and SOS2 (version 4 build 6) contains 466 samples of the same genes (Kotiang and Eslami, 2020). In this section, we applied SOS1 data for the logic gate inference with wpLogicNet, as it is less noisy. Supplementary Figure S5 and Supplementary Table S2 show the gold standard SOS regulatory interactions with 43 edges, derived from literature (Bansal *et al.*, 2006; Gardner *et al.*, 2003; Kotiang and Eslami, 2020).

**Fig. 5.**
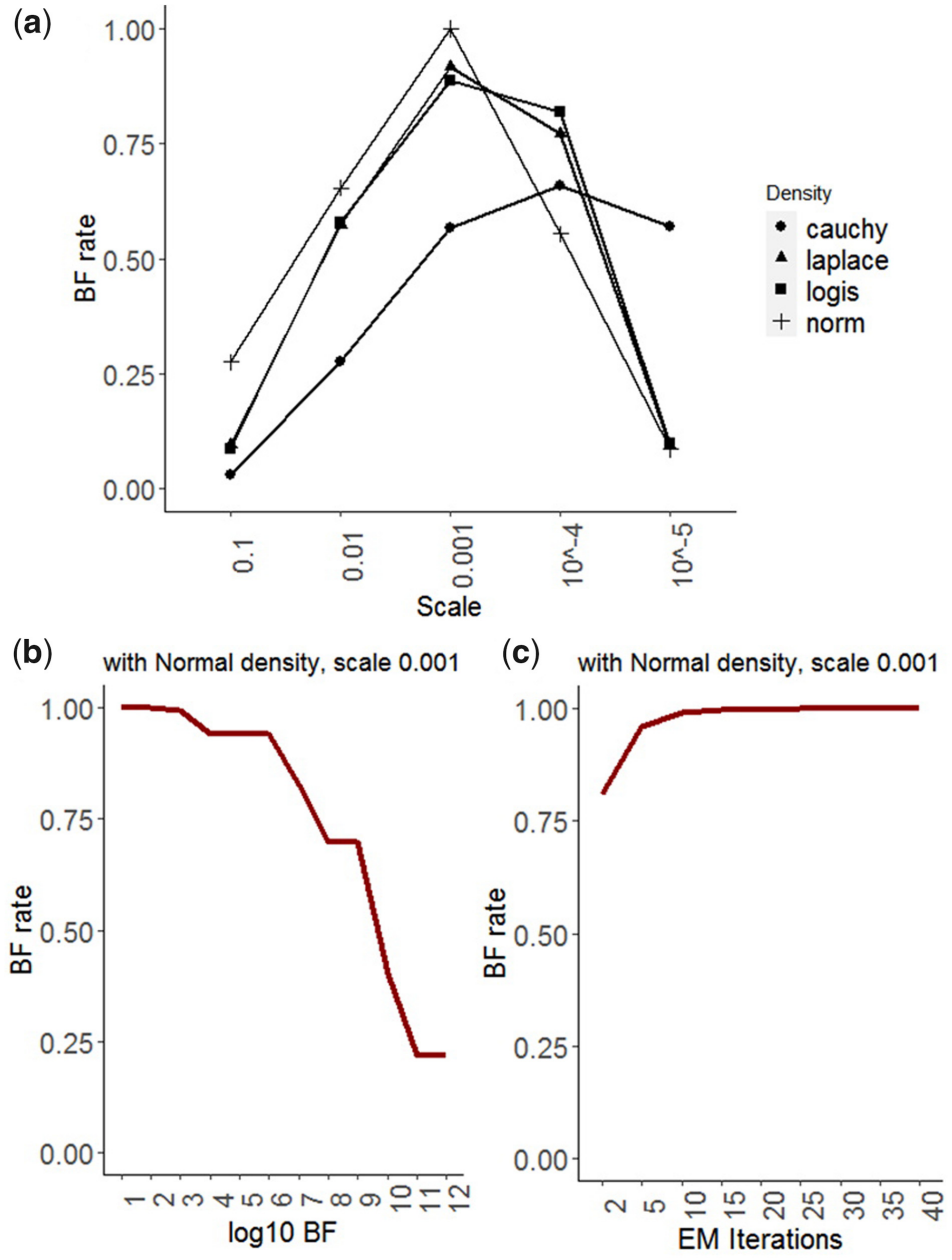
Parameter estimation in structure-aware logic gate inference in the SOS DNA-repair network. The BF rate versus (**a**) scale parameter per density, (**b**) ${\;\text{log}\;}_{10}\text{BF}$ for Normal density and (**c**) the number of required EM iterations for Normal density

Optimal parameters are first derived from models with the best BF rate. In Figure 5a, the BF rate is plotted versus scale parameters $\left\{10^{-1},10^{-2},10^{-3},10^{-4},10^{-5}\right\}$, with ${\;\text{log}\;}_{10}\text{BF}$=2 and EM iterations =10. For the majority of densities, the highest BF rate is given by scale=$10^{-3}$. In Figure 5b, the BF rate is plotted versus ${\;\text{log}\;}_{10}\text{BF}$ in the (1,12) interval, for Normal density with scale=$10^{-3}$ and EM iterations =10. In Figure 5c, the BF rate is plotted versus EM iterations, for Normal density with scale=$10^{-3}$ and ${\;\text{log}\;}_{10}\text{BF}$=2. Considering the model with the highest BF rate, we inferred the logic gates by fitting a Normal density with scale=$10^{-3}$, ${\;\text{log}\;}_{10}\text{BF}$=2 as a threshold for logic gate significance, and EM iteration =10, see Table 5. The high-priority logic combinations that generate the greatest number of observations from the target gene are also marked in bold for each gate.

**Table 5.** The inferred logic gates for the SOS DNA-repair network of *E.coli*, with wpLogicNet in structure-aware mode

Gene	$-L_{0}$	$-L_{1}$	LBF	Predicted logic gate
**recA**	14.55	3.83	10.72	$\bar{\text{lexA}}\cdot \text{ssb}\cdot \text{recF}\cdot \bar{\text{dinI}\;}\cdot \bar{\text{umuDC}}\cdot \text{rpoD }\cup$ $\bar{l\mathrm{exA}}\cdot ssb\cdot \bar{r\mathrm{ecF}}\cdot \bar{d\mathrm{inI}}\cdot \mathrm{umuDC}\cdot \bar{\mathrm{rpoD}}\cup$ $\bar{\text{lexA}}\cdot \bar{\text{ssb}}\cdot \text{recF}\cdot \bar{\text{dinI}}\cdot \bar{\text{umuDC}}\cdot \bar{\text{rpoD}}$
**lexA**	12.54	9.48	3.06	$\bar{r\mathrm{ecA}}\cdot ssb\cdot \bar{r\mathrm{ecF}}\cdot \bar{d\mathrm{inI}}\cdot \bar{u\mathrm{muDC}}\cdot r\mathrm{poD}\cup$ $\bar{\text{recA}}\cdot \bar{\text{ssb}}\cdot \bar{\text{recF}}\cdot \text{dinI}\cdot \bar{\text{umuDC}}\cdot \bar{\text{rpoD}}$
**ssb**	14.57	1.88	12.69	$\bar{\text{recA}}\cdot \bar{\text{lexA}}\cdot \bar{\text{recF}}\cdot \bar{\text{dinI}}\cdot \text{umuDC}\cdot \text{rpoD}\cup$ $\bar{\text{recA}}\cdot \bar{\text{lexA}}\cdot \text{recF}\cdot \bar{\text{dinI}}\cdot \bar{\text{umuDC}}\cdot \text{rpoD}\cup$ $\bar{r\mathrm{ecA}}\cdot l\mathrm{exA}\cdot \bar{r\mathrm{ecF}}\cdot d\mathrm{inI}\cdot \bar{u\mathrm{muDC}}\cdot \bar{r\mathrm{poD}}\cup$ $\bar{\text{recA}}\cdot \bar{\text{lexA}}\cdot \bar{\text{recF}}\cdot \bar{\text{dinI}}\cdot \text{umuDC}\cdot \bar{\text{rpoD}}\cup$ $\bar{\text{recA}}\cdot \text{lexA}\cdot \bar{\text{recF}}\cdot \bar{\text{dinI}}\cdot \bar{\text{umuDC}}\cdot \bar{\text{rpoD}}$
**recF**	18.00	8.96	9.04	$\text{ssb}\cdot \bar{\text{umuDC}}\cdot \text{rpoD}\cdot \text{rpoS}\cup$ $ssb\cdot \bar{u\mathrm{muDC}}\cdot r\mathrm{poD}\cdot \bar{r\mathrm{poS}}\cup$ $\text{ssb}\cdot \bar{\text{umuDC}}\cdot \bar{\text{rpoD}}\cdot \bar{\text{rpoS}}$
**dinI**	16.53	9.72	6.80	$\bar{r\mathrm{ecA}}\cdot \bar{l\mathrm{exA}}\cdot \bar{ssb}\cdot \bar{r\mathrm{ecF}}\cdot u\mathrm{muDC}\cdot r\mathrm{poD}\cup$ $\bar{\text{recA}}\cdot \text{lexA}\cdot \bar{\text{ssb}}\cdot \bar{\text{recF}}\cdot \bar{\text{umuDC}}\cdot \bar{\text{rpoD}}$
**umuDC**	9.51	2.00	7.51	$\text{recA}\cdot \text{lexA}\cdot \bar{\text{ssb}}\cdot \bar{\text{recF}\;}\cdot \bar{\text{dinI}}\cdot \text{rpoD}\cup$ $\bar{r\mathrm{ecA}}\cdot \bar{l\mathrm{exA}}\cdot \bar{ssb}\cdot \bar{r\mathrm{ecF}}\cdot d\mathrm{inI}\cdot r\mathrm{poD}\cup$ $\bar{\text{recA}}\cdot \text{lexA}\cdot \text{ssb}\cdot \bar{\text{recF}\;}\cdot \bar{\text{dinI}}\cdot \bar{\text{rpoD}}$
**rpoD**	14.12	4.50	9.62	$\bar{\text{recA}}\cdot \bar{\text{lexA}}\cdot \bar{\text{ssb}}\cdot \text{recF}\cdot \text{dinI}\cdot \text{umuDC}\cdot \bar{\text{rpoH}}\cup$ $\bar{\text{recA}}\cdot \text{lexA}\cdot \bar{\text{ssb}}\cdot \bar{\text{recF}}\cdot \text{dinI}\cdot \text{umuDC}\cdot \bar{\text{rpoH}}\cup$ $\text{recA}\cdot \bar{\text{lexA}}\cdot \text{ssb}\cdot \text{recF}\cdot \bar{\text{dinI}}\cdot \bar{\text{umuDC}}\cdot \bar{\text{rpoH}}\cup$ $\bar{r\mathrm{ecA}}\cdot l\mathrm{exA}\cdot ssb\cdot \bar{r\mathrm{ecF}}\cdot \bar{d\mathrm{inI}}\cdot \bar{u\mathrm{muDC}}\cdot \bar{r\mathrm{poH}}$
**rpoH**	18.00	18.00	0.00	—
**rpoS**	18.00	15.98	2.02	**rpoD**

*Note*: For each logic gate, the high-priority logic combinations, i.e. those that generate the greatest number of observations from the corresponding target gene, are highlighted in bold. $-{\;\text{Log}\;}_{10}L_{0}$,**−*L*_0_**; $-{\;\text{log}\;}_{10}L_{1}$, **−*L*_1_**; ${\;\text{Log}\;}_{10}\text{BF}$, **LBF**.

### 3.3 Directed edge and logic gate inference

**Fig. 6.**
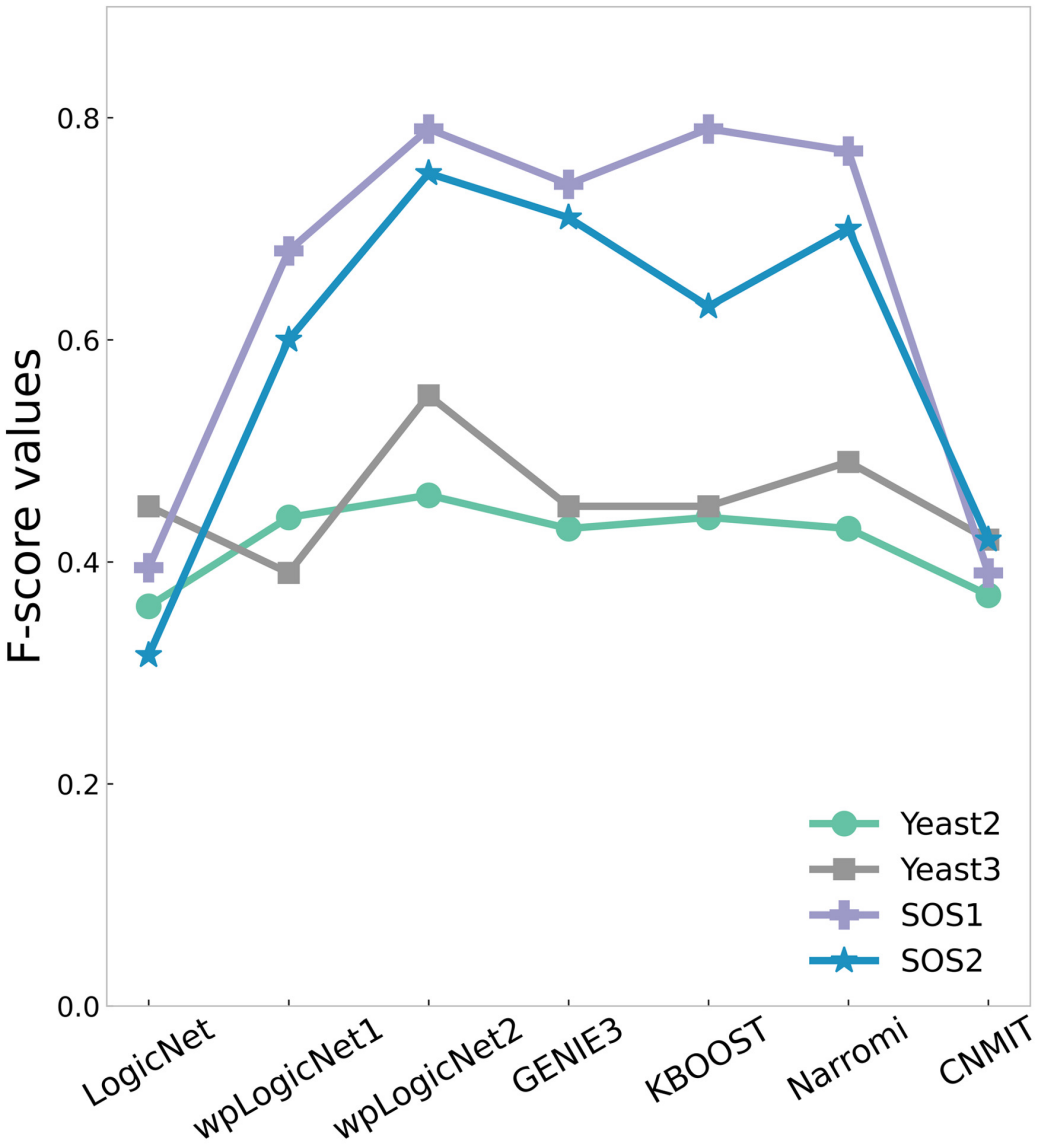
*F*-scores of wpLogicNet (top-logic):wpLogicNet1, wpLogicNet (top-edges):wpLogicNet2 and five well-known methods for learning DREAM3 Challenge networks Yeast2 and Yeast3, with 10 genes and 25 and 22 gold standard directed edges, respectively, and SOS DNA-repair network with 9 genes and 43 edges (9 and 466 microarray gene expression samples for SOS1 and SOS2 datasets). LogicNet and wpLogicNet are logic-based models to infer directed edges and logic gates among regulators, simultaneously. As LogicNet is very slow in evaluating the candidate RG sets, it was fitted considering logic gates with up to k=3 regulators. wpLogicNet is fitted considering logic gates with up to k=8 regulators. GENIE3, KBOOST, Narromi and CNMIT do not infer logic gates, i.e. only infer directed edges

wpLogicNet also infers a directed GRN structure together with logic gates for GRNs with unknown structure. In this section, we use both simulated (DREAM project) and real data (SOS DNA-repair network). The DREAM project is an *in silico* network challenge introduced in 2006 (Marbach *et al.*, 2010). We use two steady-state datasets *Yeast2* and *Yeast3* in DREAM3 that have 10 genes with 25 and 22 gold standard directed edges, respectively. Supplementary Figures S5–S7 show the gold standard structure of these networks. The performance of wpLogicNet (top-logics) and wpLogicNet (top-edges) are benchmarked against five well-known algorithms in inferring the directed edges (Fig. 6). For evaluating the results, we use true positive, true negative, false positive, false negative, recall, FPR, specificity, precision, accuracy and *F*-score (Altman and Bland, 1994). For threshold-dependent algorithms, i.e. GENIE3, KBOOST, Narromi and CNMIT, we have incrementally increased the threshold in the (0,1) interval and selected the optimal threshold reaching the maximum *F*1-score to report the result per algorithm (Mahmoodi *et al.*, 2021). See Supplementary Table S3 for the optimal thresholds applied to each algorithm. Summarized results in Supplementary Table S4 and Figure 6 show that wpLogicNet (top-edges) consistently performs better than all other algorithms in terms of *F*-score. As shown in Supplementary Table S5, the running time of LogicNet is noticeably longer than wpLogicNet, although LogicNet was fitted considering logic gates with up to k=3 RGs (compared to k=8 in wpLogicNet).

## 4 Discussion

As shown in Section 3.1, wpLogicNet can be applied to reconstruct TF–gene interaction networks and to infer logic gates among multiple TFs. wpLogicNet in a structure-aware mode infers the gates among RGs or regulatory TFs that control their target, where directed edges are specified *a priori*, as shown in the SOS DNA-repair network in Section 3.2. Moreover, in the GRNs with unknown structure, wpLogicNet can be applied for the simultaneous inference of the directed edges and logic gates, as shown in Section 3.3.wpLogicNet proved to be accurate and reliable in inferring directed edges from TFs to target operons and logic gates among TFs in DORs. Moreover, wpLogicNet is superior to the Loregic for the logic gate inference in structure-aware mode. However, as wpLogicNet is dealing with noisy (non-binary) data, a drop in AUPRs is more obvious in wpLogicNet than in Loregic with an increase in noise. The difference between wpLogicNet and Loregic accuracies, in the logic gate inference, can be attributed to the extent of information loss in the data binarization process.

Computational complexity in the logic-based models arises from (i) selection of candidate RG sets and (ii) evaluation of all possible logic gates per candidate RG set. To reduce the computational complexities due to (i), LogicTRN applies external hint data, e.g. TF-binding site data to narrow down the candidate regulatory TF pool. Other tools, such as Loregic, work with an *a priori*-specified network structure. To reduce the computational complexities due to (ii), LogicTRN and Loregic infer logic gates between only two TFs or genes. Moreover, due to the modeling limitations, LogicTRN is restricted to inferring six possible logic gates between two TFs. In contrast to these tools, wpLogicNet offers an algorithmic approach to infer logic gates among RG sets of any size.

Moreover, compared to LogicNet, wpLogicNet considerably lowers the time-complexity of the logic gate inference, allowing it to reconstruct larger GRNs with more complex regulatory relationships including more genes in a gate. wpLogicNet in structure-aware mode requires only one likelihood calculation for the gate inference per target gene. For example in the SOS DNA-repair network for recA with six regulators (lexA, ssb, recF, dinI, umuDC and rpoD), wpLogicNet and LogicNet calculate 1 and $2^{2}$^^6^^ likelihoods for the logic gate inference, respectively.

## 5 Conclusion

In this article, we introduce an intriguing GRN reconstruction method—called wpLogicNet—to represent the gene regulatory process by logic gates based on continuous gene expressions. Studying the nature of gene interactions suggests that the gene regulatory process has all the hallmarks of a logic system in which a target gene is regulated by a logic gate among its regulators (AkhavanAghdam *et al.*, 2016; Fauré *et al.*, 2006; Krumsiek *et al.*, 2011; Moignard *et al.*, 2015; Schlatter *et al.*, 2009; Szczurek *et al.*, 2009). However, considering such logical relationships among genes has received little attention in prior GRN reconstruction studies. This oversight prevents the existing methods from fully depicting the true gene regulation process. High computational complexity is the major cause of this disregard. Specifically, many logic-based models, e.g. LogicTRN and Loregic, are restricted to deciphering the logic between two RGs or TFs. To overcome this drawback, we introduce a novel approach to define all possible logic gates among a sensible number of RGs without any restrictions.

In contrast to the existing logic-based tools, wpLogicNet does not need an *a priori*-specified network topology for GRN reconstruction. However, given a known GRN structure, wpLogicNet in structure-aware mode can infer logic gates among RGs for regulating their target. This can be applied to the further characterization of the biological networks and their functions, e.g. in gene–gene or TF–gene networks with known structures.

Although the reported logic gates in this article are optimum ones, corresponding to the maximum significant likelihood, wpLogicNet determines a family of logic gates in which each member can be considered as a candidate gate among genes. However, quality assessment for all candidate logic gates is not possible because of the limited data about regulatory relationships and the high cost of performing new wet-lab experiments. To further narrow down this family of candidate logic gates, extra knowledge, e.g. Knock-Out or Over-Expression experiment, is needed to ascertain the ON or OFF states of logic combinations with high certainty.

Integrating other omics data, such as proteins, or cis-elements, which act as the TF-binding sites and control the spatial and temporal expression of nearby genes, can also narrow down the logic gate families and uncertainties in network inference. Consequently, these developments in biological information could be the basis for future improvements in gene expression modeling.

## Supplementary Material

btad072_Supplementary_Data

## Data Availability

The data underlying this article are available in github repository, https://github.com/CompBioIPM/wpLogicNet.

## References

[btad072-B1] Aghdam R. et al (2015) CN: a consensus algorithm for inferring gene regulatory networks using the SORDER algorithm and conditional mutual information test. Mol. Biosyst., 11, 942–949. 25607659 10.1039/c4mb00413b

[btad072-B2] AkhavanAghdam Z. et al (2016) Dynamic control of gene regulatory logic by seemingly redundant transcription factors. Elife, 5, e18458. 27690227 10.7554/eLife.18458 PMC5047750

[btad072-B3] Aldridge B. et al (2009) Fuzzy logic analysis of kinase pathway crosstalk in TNF/EGF/insulin-induced signaling. PLoS Comput. Biol., 5, e1000340. 19343194 10.1371/journal.pcbi.1000340 PMC2663056

[btad072-B4] Alizad-Rahvar A.R. , Sadeghi M. (2018) Ambiguity in logic-based models of gene regulatory networks: an integrative multi-perturbation analysis. PLoS One, 13, e0206976. 30458000 10.1371/journal.pone.0206976 PMC6245684

[btad072-B5] Altman D.G. , Bland J.M. (1994) Diagnostic tests. 1: sensitivity and specificity. BMJ, 308, 1552. 8019315 10.1136/bmj.308.6943.1552 PMC2540489

[btad072-B6] Bansal M. et al (2006) Inference of gene regulatory networks and compound mode of action from time course gene expression profiles. Bioinformatics, 22, 815–822. 16418235 10.1093/bioinformatics/btl003

[btad072-B7] Barman S. , Kwon Y.-K. (2017) A novel mutual information-based Boolean network inference method from time-series gene expression data. PLoS One, 12, e0171097. 28178334 10.1371/journal.pone.0171097 PMC5298315

[btad072-B8] Berger J. , Pericchi L. (2015) Bayes Factors. John Wiley & Sons, Ltd., USA. pp. 1–14. 10.1002/9781118445112.stat00224.pub2.

[btad072-B9] Collombet S. et al (2017) Logical modeling of lymphoid and myeloid cell specification and transdifferentiation. Proc. Natl. Acad. Sci. USA, 114, 5792–5799. 28584084 10.1073/pnas.1610622114 PMC5468615

[btad072-B10] Fauré A. et al (2006) Dynamical analysis of a generic Boolean model for the control of the mammalian cell cycle. Bioinformatics, 22, e124–e131. 16873462 10.1093/bioinformatics/btl210

[btad072-B11] Gardner T. et al (2003) Inferring genetic networks and identifying compound mode of action via expression profiling. Science, 301, 102–105. 12843395 10.1126/science.1081900

[btad072-B12] Goodman L. A. (1960) On the exact variance of products. J. Am. Stat. Assoc., 55, 708–713.

[btad072-B13] Huynh-Thu V. et al (2010) Inferring regulatory networks from expression data using tree-based methods. PLoS One, 5, e12776. 20927193 10.1371/journal.pone.0012776 PMC2946910

[btad072-B14] Iglesias-Martinez L. et al (2021) KBoost: a new method to infer gene regulatory networks from gene expression data. Sci. Rep., 11, 1–13. 34326402 10.1038/s41598-021-94919-6 PMC8322418

[btad072-B15] Jiang C. , Pugh B.F. (2009) Nucleosome positioning and gene regulation: advances through genomics. Nat. Rev. Genet., 10, 161–172. 19204718 10.1038/nrg2522 PMC4860946

[btad072-B16] Kotiang S. , Eslami A. (2020) A probabilistic graphical model for system-wide analysis of gene regulatory networks. Bioinformatics, 36, 3192–3199. 32096828 10.1093/bioinformatics/btaa122

[btad072-B17] Krumsiek J. et al (2011) Hierarchical differentiation of myeloid progenitors is encoded in the transcription factor network. PLoS One, 6, e22649. 21853041 10.1371/journal.pone.0022649 PMC3154193

[btad072-B18] Mahmoodi S. et al (2021) An order independent algorithm for inferring gene regulatory network using quantile value for conditional independence tests. Sci. Rep., 11, 1–15. 33828122 10.1038/s41598-021-87074-5 PMC8027014

[btad072-B19] Malekpour S. et al (2020) LogicNet: probabilistic continuous logics in reconstructing gene regulatory networks. BMC Bioinformatics, 21, 1–21. 32690031 10.1186/s12859-020-03651-x PMC7372900

[btad072-B20] Marbach D. et al (2010) Revealing strengths and weaknesses of methods for gene network inference. Proc. Natl. Acad. Sci. USA, 107, 6286–6291. 20308593 10.1073/pnas.0913357107 PMC2851985

[btad072-B21] Moignard V. et al (2015) Decoding the regulatory network of early blood development from single-cell gene expression measurements. Nat. Biotechnol., 33, 269–276. 25664528 10.1038/nbt.3154 PMC4374163

[btad072-B22] Morris M. et al (2011) Training signaling pathway maps to biochemical data with constrained fuzzy logic: quantitative analysis of liver cell responses to inflammatory stimuli. PLoS Comput. Biol., 7, e1001099. 21408212 10.1371/journal.pcbi.1001099 PMC3048376

[btad072-B23] Prill R. et al (2010) Towards a rigorous assessment of systems biology models: the DREAM3 challenges. PLoS One, 5, e9202. 20186320 10.1371/journal.pone.0009202 PMC2826397

[btad072-B24] Pušnik Ž. et al (2022) Review and assessment of Boolean approaches for inference of gene regulatory networks. Heliyon, 8, e10222. 36033302 10.1016/j.heliyon.2022.e10222 PMC9403406

[btad072-B25] Ronen M. et al (2002) Assigning numbers to the arrows: parameterizing a gene regulation network by using accurate expression kinetics. Proc. Natl. Acad. Sci. USA, 99, 10555–10560. 12145321 10.1073/pnas.152046799 PMC124972

[btad072-B26] Schlatter R. et al (2009) On/off and beyond-a Boolean model of apoptosis. PLoS Comput. Biol., 5, e1000595. 20011108 10.1371/journal.pcbi.1000595 PMC2781112

[btad072-B27] Shen-Orr S. et al (2002) Network motifs in the transcriptional regulation network of *Escherichia coli*. Nat. Genet., 31, 64–68. 11967538 10.1038/ng881

[btad072-B28] Szczurek E. et al (2009) Elucidating regulatory mechanisms downstream of a signaling pathway using informative experiments. Mol. Syst. Biol., 5, 287. 19584836 10.1038/msb.2009.45 PMC2724975

[btad072-B29] Touré V. et al (2021) The status of causality in biological databases: data resources and data retrieval possibilities to support logical modeling. Brief. Bioinform., 22, bbaa390. 33378765 10.1093/bib/bbaa390 PMC8294520

[btad072-B30] Wang D. et al (2015) Loregic: a method to characterize the cooperative logic of regulatory factors. PLoS Comput. Biol., 11, e1004132. 25884877 10.1371/journal.pcbi.1004132 PMC4401777

[btad072-B31] Yan B. et al (2017) An integrative method to decode regulatory logics in gene transcription. Nat. Commun., 8, 1–12. 29051499 10.1038/s41467-017-01193-0 PMC5715098

[btad072-B32] Zhang X. et al (2013) NARROMI: a noise and redundancy reduction technique improves accuracy of gene regulatory network inference. Bioinformatics, 29, 106–113. 23080116 10.1093/bioinformatics/bts619

