# Correction to: wpLogicNet: logic gate and structure inference in gene regulatory networks

**DOI:** 10.1093/bioinformatics/btad304

**Published:** 2023-05-17

This is a correction to: Seyed Amir Malekpour, Maryam Shahdoust, Rosa Aghdam, Mehdi Sadeghi, wpLogicNet: logic gate and structure inference in gene regulatory networks, *Bioinformatics*, Volume 39, Issue 2, February 2023, https://doi.org/10.1093/bioinformatics/btad072

In the originally published version of this manuscript, the last author’s affiliation was presented incorrectly. The correct affiliation for Professor Mehdi Sadeghi is as follows: Department of Medical Genetics, National Institute for Genetic Engineering and Biotechnology, Tehran 1497716316, Iran.

This error has been corrected online.

