# Supplementary material for: wpLogicNet: logic gate and structure inference in gene regulatory networks
Source: Bioinformatics. 2023 Feb 15;39(2):btad072. doi: 10.1093/bioinformatics/btad072 (PMC9936836; doi:10.1093/bioinformatics/btad072)
Supplement: btad072_Supplementary_Data [file btad072_supplementary_data.pdf]

wpLogicNet: logic gate and structure inference in gene regulatory networks

SEYED AMIR MALEKPOUR[1], MARYAM SHAHDOUST[1], ROSA AGHDAM[1,2], AND MEHDI SADEGHI[1]

[1] *School of Biological Sciences, Institute for Research in Fundamental Sciences (IPM), Tehran 19395-5746, Iran*
[2] *Wisconsin Institute for Discovery , University of Wisconsin-Madison, Madison, WI 53715, USA*

DATA NORMALIZATION

To normalize the gene profile matrix, where rows are samples and columns are genes, the expression profile is transformed into an interval $(0^+, 1-\delta)$ using Eq (S1):

$$\min(1-\ \delta\ ,\ \frac{g_i^s - \min_s + \alpha}{max_s - \min_s + \alpha}\ ), \tag{S1}$$

Where g_i^s denotes the profile level of gene i for s^{th} sample, and min_s and max_s are the minimum and maximum observed expressions for the sth sample in the profile matrix, respectively. Here, δ and α are small positive values such as 0.01, introduced to avoid obtaining the absolute 0 or 1 for normalized gene profile level (see Fig. S1).

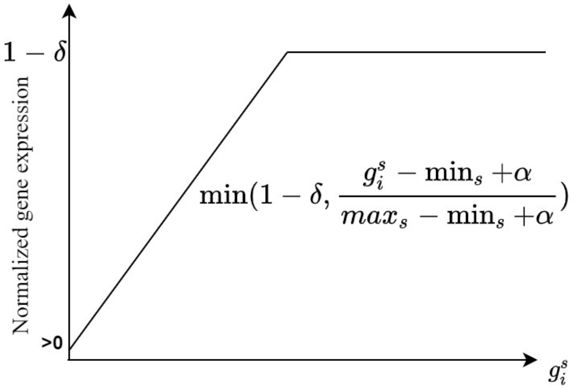

Fig. S1. Gene Expression Normalization Method. g_i^s is the expression value of gene i for s^{th} sample. The minimum and maximum expression values for s^{th} sample are denoted by min_s and max_s, respectively. The notations δ and α are small positive values, e.g., 0.01.

ON DERIVING EQUATION 6 OF THE MAIN TEXT

$$\frac{\partial}{\partial \omega_v}\left[\sum_{v=0}^{2^k-1}\sum_{s=1}^{n} log(\omega_v)p(v|t_s,\Theta^m) + \lambda(\sum_{v=0}^{2^k-1}\omega_v - 1)\right] = 0 \tag{S2}$$

By taking the derivative of the above eq. with respect to ω_v,

$$\sum_{s=1}^{n} p\left(v \mid t_s, \Theta^m\right) + \omega_v\lambda = 0 \tag{S3}$$

Summing both sizes over v gives:

$$\sum_{s=1}^{n}\sum_{v=0}^{2^k-1} p\left(v \mid t_s, \Theta^m\right) + \lambda = 0 \tag{S4}$$

and

$$\sum_{s=1}^{n} \sum_{v=0}^{2^k-1} \frac{\omega_v p\left(t_s \mid v, \Theta^m\right)}{p\left(t_s \mid \Theta^m\right)} + \lambda = 0 \tag{S5}$$

Then we get that $\lambda = -n$ and update ω_v in EM algorithm with following equation:

$$\omega_v^{m+1} = \frac{1}{n} \sum_{s=1}^{n} p\left(v \mid t_s, \Theta^m\right) = \frac{1}{n} \sum_{s=1}^{n} \frac{\omega_v^m p\left(t_s \mid v\right)}{\sum_{v'=0}^{2^k-1} \omega_{v'}^m p\left(t_s \mid v'\right)} \tag{S6}$$

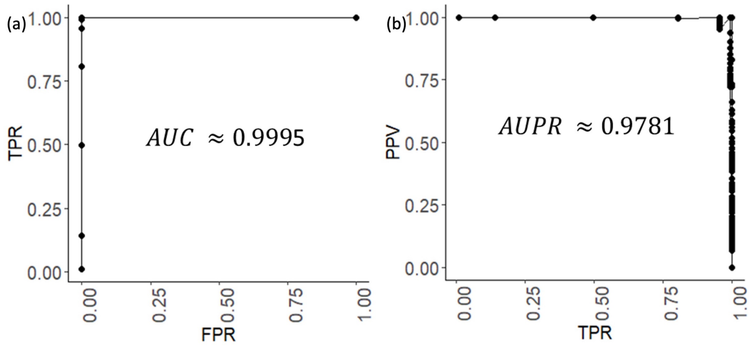

Fig. S2. (a) Area Under the receiver operating characteristic Curve (AUC) and (b) Area Under the precision-recall curve (AUPR), for the logic gate inference in small DORs with 10 TFs and 7 genes, with fitting a Normal density, scale=0.005, $\log_{10}\text{BF} = 2$, EM iterations=10 and $\sigma = 0.01$ as the noise level for all TFs.

Table S1. The running time of wpLogicNet in the logic gate inference among TFs, in a small DOR with 10 TFs and 7 operons. The running time is reported considering logic gates with up to k candidate regulatory TFs in a gate.

k[a]	Number of candidate TF sets[b]	Run time in seconds[c]
1	10	0.52
2	55	0.99
3	175	4.03
4	385	14.36
5	637	37.46
6	847	75.69
7	967	119.70
8	1012	151.52
9	1022	167.32
10	1023	169.20

[a] This is the maximum number of candidate regulatory TFs, in a gate.
[b] In wpLogicNet, considering up to k regulatory TFs in a gate, likelihoods are evaluated for $\sum_{i=1}^{k} \binom{10}{i}$ candidate TF sets, per target gene. In our Bayesian model, logic gates are then inferred a posteriori, per candidate TF set.
[c] Scale parameter, BF threshold, and number of EM iterations=10 are fixed, in fitting wpLogicNet.

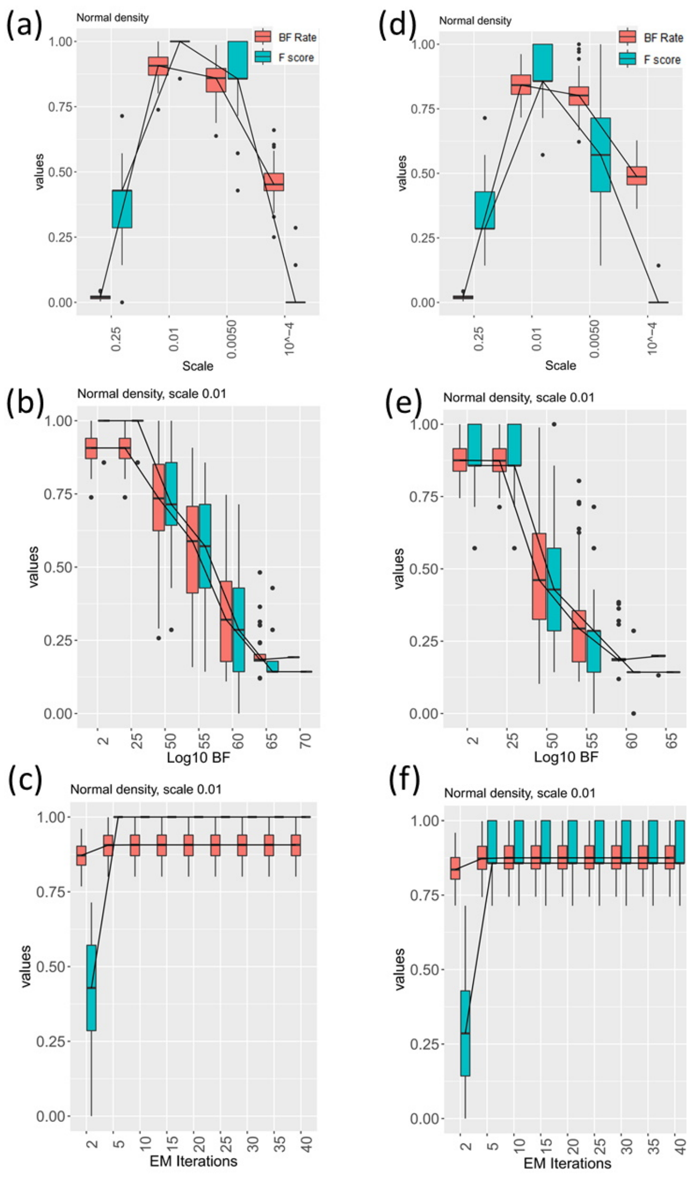

Fig. S3. Parameter setting and F-score for the logic gate inference in a small DOR with 10 TFs and 7 genes. For noise levels $\sigma = 0.02$ (a,b,c), $\sigma = 0.03$ (d,e,f), with sample size 20 and 100 repeats of whole simulation study, as described in section 3.1. (a) and (d) with fitting a Normal density over a range of scale parameters $\{0.25, 0.01, 0.005, 10^{-4}\}$, $\text{Log}_{10}\text{BF} = 2$, and EM iterations=10. (b) and (e) with fitting a Normal density, scale=0.01, Log_{10}BF in the (2-70) interval, and EM iterations=10. (c) and (f) with fitting a Normal density, scale=0.01, $\text{Log}_{10}\text{BF} = 2$, and EM iterations in the (2-40) interval.

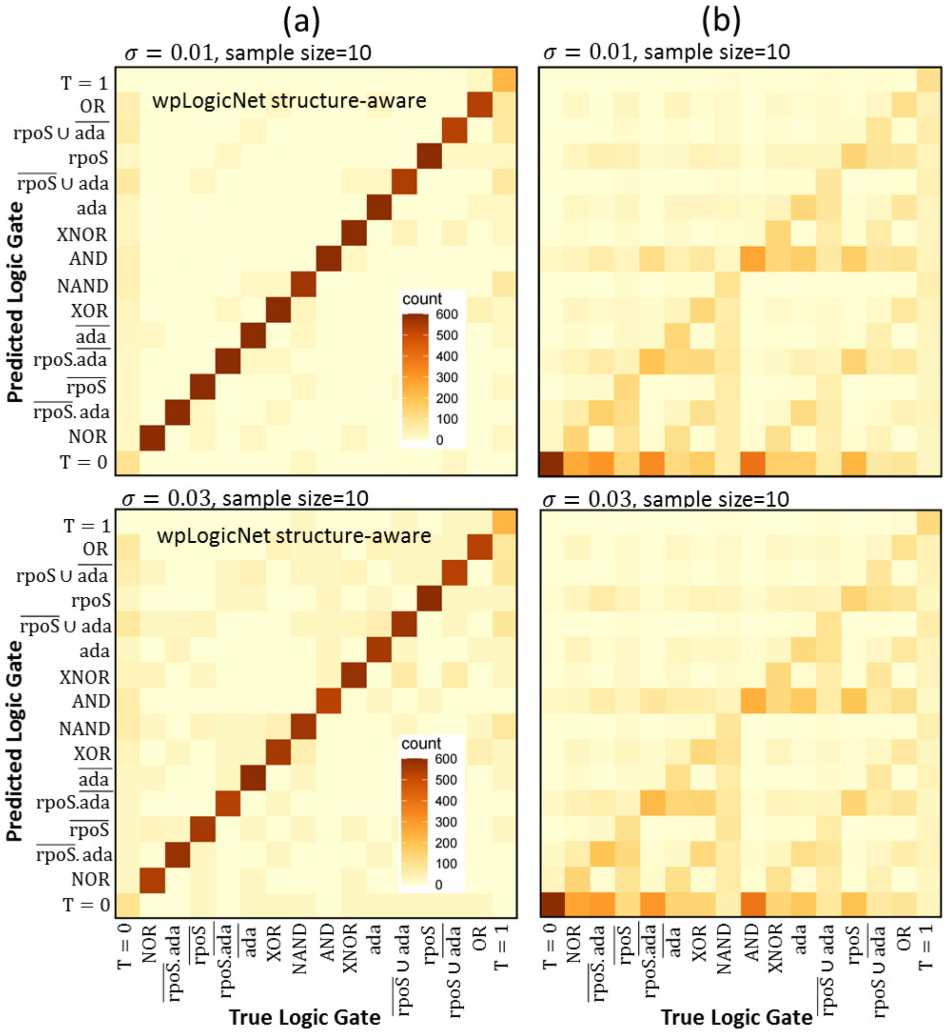

Fig. S4. wpLogicNet in structure-aware mode is compared to Loregic. Frequencies (in orange) of the true vs. predicted logic gate classes are shown for wpLogicNet (a) and Loregic (b), for two noise levels σ= 0.01, 0.03. For each simulated logic gate, both tools take in the expression profile and directed edges from rpoS and ada –> alkA, to predict the gate. Counts are based on 10,000 simulated logic gates, and 10 samples from TFs-alkA, per gate.

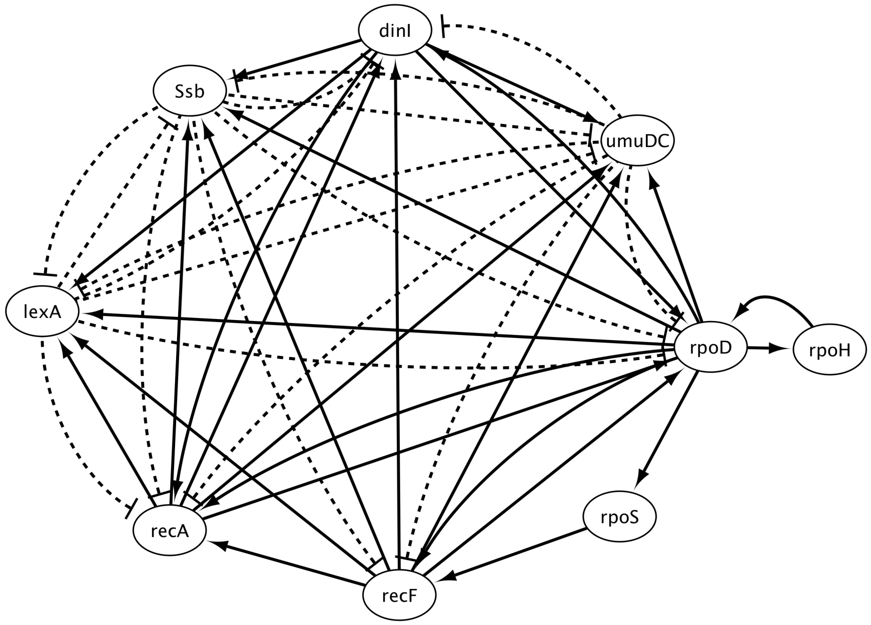

Fig. S5. Gold Standard for SOS DNA-repair network with 9 genes and 43 edges. SOS network includes two mediators of the SOS response (lexA and recA), four other regulatory genes (ssb, recF, dinI, and umuDC) involved in the SOS response, and three sigma factor genes (rpoD, rpoH, and rpoS) whose regulation plays important role in the SOS response.

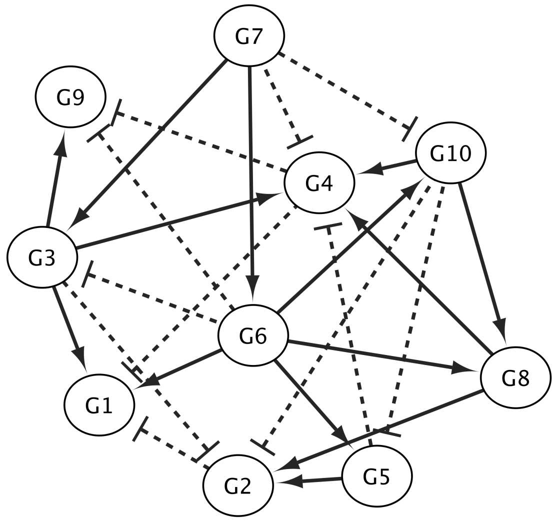

Fig. S6. Gold standard for Yeast2. Yeast2 is steady-state dataset with 10 genes and 25 gold standard edges.

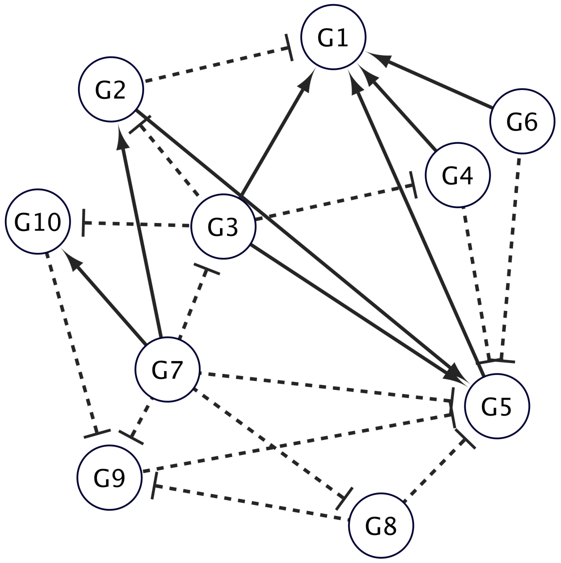

Fig. S7. Gold standard for Yeast 3. Yeast3 is steady-state dataset with 10 genes and 22 gold standard edges.

Table S2. Gold standard for SOS DNA-repair network with 9 genes and 43 edges. An activatory, inhibitory, or no regulatory input from the gene in the column to the gene in the row is indicated by +1, -1, or 0.

genes	recA	lexA	Ssb	recF	dinI	umuDC	rpoD	rpoH	rpoS
recA	1	-1	-1	1	1	-1	1	0	0
lexA	1	-1	-1	1	1	-1	1	0	0
Ssb	1	-1	-1	1	1	-1	1	0	0
recF	0	0	-1	0	0	-1	1	0	1
dinI	1	-1	-1	1	1	-1	1	0	0
umuDC	1	-1	-1	1	1	-1	1	0	0
rpoD	1	-1	-1	1	1	-1	1	1	0
rpoH	0	0	0	0	0	0	1	1	0
rpoS	0	0	0	0	0	0	1	0	1

Table S3. The optimal thresholds applied to the threshold-dependent algorithms GENIE3, KBOOST, Narromi, and CNMIT, in the directed edge inference of SOS1, SOS2, Yeast2, and Yeast3 networks.

Network	GENIE3	KBOOST	Narromi	CNMIT
SOS1	0.01829	0.00061	0.02429	$\theta_1 : (0, 0.05), \theta_2 = 0.7$
SOS2	0.05943	0.00080	0.15299	$\theta_1 : (0, 0.05), \theta_2 = 0.7$
Yeast2	0.03975	0.00074	0.47264	$\theta_1 : (0, 0.05), \theta_2 = 0.7$
Yeast3	0.04746	0.00229	0.11631	$\theta_1 : (0, 0.05), \theta_2 = 0.7$

Table S4. Classification results of well-known algorithms for SOS1, SOS2, Yeast2 and Yeast3. The best results are indicated in bold. Specificity:Spe, Precision:Pre, Accuracy:Acc, F_{score}:F_S, wpLogicNet (top-logics):wpLogicNet1, wpLogicNet (top-edges):wpLogicNet2

	Method	TP	TN	FP	FN	Rec	FPR	Spe	Pre	Acc	F_s
**SOS1**	wpLogicNet1	28	18	11	15	0.65	0.38	0.62	0.72	0.64	0.68
	wpLogicNet2	**43**	6	23	**0**	**1**	0.79	0.21	0.65	0.68	**0.79**
	LogicNet	15	11	10	36	0.29	0.48	0.52	0.60	0.21	0.39
	GENE3	42	0	29	1	0.98	1	0	0.59	0.58	0.74
	KBOOST	42	8	21	1	0.98	0.72	0.28	0.67	**0.69**	**0.79**
	Narromi	42	5	24	1	0.98	0.83	0.17	0.64	0.65	0.77
	CNMIT	11	**26**	**3**	32	0.26	**0.10**	**0.90**	**0.79**	0.51	0.39
**SOS2**	wpLogicNet1	27	9	20	16	0.63	0.69	0.31	0.57	0.50	0.60
	wpLogicNet2	**42**	2	27	**1**	**0.98**	0.93	0.07	0.61	**0.61**	**0.75**
	LogicNet	12	8	13	39	0.23	0.62	0.38	0.48	0.17	0.32
	GENE3	34	10	19	9	0.79	0.66	0.34	0.64	**0.61**	0.71
	KBOOST	28	11	18	15	0.65	0.62	0.38	0.61	0.54	0.63
	Narromi	36	5	24	7	0.84	0.83	0.17	0.60	0.57	0.70
	CNMIT	13	**23**	**6**	30	0.30	**0.21**	**0.79**	**0.68**	0.50	0.42
**Yeast2**	wpLogicNet1	17	30	35	8	0.68	0.54	0.46	0.33	0.52	0.44
	wpLogicNet2	**18**	29	36	**7**	**0.72**	0.55	0.45	0.33	0.52	**0.46**
	LogicNet	10	45	20	15	0.40	0.31	0.69	0.33	0.61	0.36
	GENE3	**23**	5	60	**2**	**0.92**	0.92	0.08	0.28	0.31	0.43
	KBOOST	**23**	8	57	**2**	**0.92**	0.88	0.12	0.29	0.34	0.44
	Narromi	21	13	52	4	0.84	0.80	0.20	0.29	0.38	0.43
	CNMIT	10	**46**	**19**	15	0.40	**0.29**	**0.71**	**0.34**	**0.62**	0.37
**Yeast3**	wpLogicNet1	14	32	36	8	0.64	0.53	0.47	0.28	0.51	0.39
	wpLogicNet2	20	37	31	2	0.91	0.46	0.54	0.39	0.63	0.55
	LogicNet	11	52	16	11	0.50	0.24	0.71	0.41	0.70	0.45
	GENE3	**21**	18	50	**1**	**0.95**	0.74	0.26	0.3	0.43	0.45
	KBOOST	12	**53**	**15**	10	0.55	**0.22**	**0.78**	0.44	**0.72**	0.49
	Narromi	20	15	53	2	0.91	0.78	0.22	0.27	0.39	0.42
	CNMIT	12	45	23	10	0.55	0.34	0.66	0.34	0.63	0.42

Table S5. The running times for wpLogicNet, LogicNet, GENIE3, KBOOST, Narromi and CNMIT. The running times are in hours:minutes:seconds orders. For example, 01:21:48 shows 1 hours, 21 minutes and 48 seconds. *stands for logic-based models to infer directed edges and logic gates among regulators, simultaneously. +stands for threshold-dependent models that can only infer directed edges in GRN. The running time of LogicNet is noticeably longer than wpLogicNet, although it was fitted considering logic gates with up to $k = 3$ RGs. wpLogicNet is fitted considering logic gates with up to $k = 8$ RGs. Benchmarking is done in Mac M1 on a 10 core/3.2 GHz, with 500GB RAM.

Network	wpLogicNet*	LogicNet*	GENIE3+	KBOOST+	Narromi+	CNMIT+
SOS1	00:00:29	00:00:41	00:00:17	00:00:16	00:00:17	00:28:53
SOS2	00:17:16	00:45:63	00:00:13	00:00:13	00:00:41	01:21:48
Yeast2	00:02:33	00:02:36	00:00:12	00:00:17	00:00:21	00:30:23
Yeast3	00:02:13	00:02:66	00:00:12	00:00:18	00:00:25	00:32:33
